# Safety Planning vs Standard Care for Suicide Prevention After Pretrial Jail Detention: A Randomized Clinical Trial

**DOI:** 10.1001/jamanetworkopen.2025.43156

**Published:** 2025-11-10

**Authors:** Lauren M. Weinstock, Richard N. Jones, Ted R. Miller, Gregory K. Brown, Sarah A. Arias, Hannah R. Graves, Ivan W. Miller, Louis Cerbo, Julie Rexroth, Holly Fitting, Danis Russell, Sheryl Kubiak, Michael D. Stein, Christopher Matkovic, Shirley Yen, Brandon A. Gaudiano, Jennifer E. Johnson

**Affiliations:** 1 Department of Psychiatry and Human Behavior, The Warren Alpert Medical School, Brown University, Providence, Rhode Island; 2 Pacific Institute for Research and Evaluation, Silver Spring, Maryland; 3 Department of Psychiatry, Perelman School of Medicine, University of Pennsylvania, Philadelphia; 4 Psychosocial Research Program, Butler Hospital, Providence, Rhode Island; 5 Department of Behavioral Healthcare, Developmental Disabilities, and Hospitals, State of Rhode Island, Cranston; 6 Genesee County Jail, Flint, Michigan; 7 Family Reentry, Community Resources for Justice, Bridgeport, Connecticut; 8 Genesee Health System, Flint, Michigan; 9 School of Social Work, Wayne State University, Detroit, Michigan; 10 Boston University School of Public Health, Boston, Massachusetts; 11 Psychiatry Emergency Service, Rhode Island Hospital, Providence; 12 Department of Psychiatry, Beth Israel Deaconess Medical Center, Boston, Massachusetts; 13 Department of Psychiatry, Harvard Medical School, Boston, Massachusetts; 14 Charles Stewart Mott Department of Public Health, College of Human Medicine, Michigan State University, Flint

## Abstract

**Importance:**

In the US, 1 in 5 adult suicide decedents has spent at least 1 night in jail in the year prior to death.

**Objective:**

To evaluate the effectiveness of the Safety Planning Intervention (SPI) with telephone follow-up as an adjunct to enhanced standard care (ESC) compared with ESC alone for reducing suicide events in the 12 months following release from pretrial jail detention.

**Design, Setting, and Participants:**

This randomized clinical trial included individuals with past 30-day suicide risk (suicide ideation with intent and/or suicide attempt) in pretrial jail detention who were recruited from 2 jails from May 11, 2016, to November 13, 2018, with a 12-month follow-up after release. Data analysis was completed from April 2023 to May 2025.

**Interventions:**

All participants received ESC. The SPI included a safety planning session in jail followed by 4 to 8 telephone calls 6 months after jail release.

**Main Outcomes and Measures:**

The primary outcome was the number of suicide events (a composite of suicide attempts and behaviors, suicide-related hospitalizations, and suicide deaths). Secondary outcomes included the number of suicide attempts, weeks and severity of active suicidal ideation, time to first suicide event, psychiatric symptoms, functioning, and hypothesized mediators. Suicidal ideation and behaviors were assessed using the Columbia-Suicide Severity Rating Scale, the Longitudinal Interval Follow-Up Evaluation, and record reviews from area hospitals. Deaths were identified through hospital, state, and national death records. Hospitalizations were measured with the Treatment History Interview and hospital records.

**Results:**

Of 800 participants randomized in jail, 655 (mean [SD] age, 33.0 [10.4] years; 473 males [72%]) were released to the community and included in analyses. Of those, 593 (91%) completed at least 1 follow-up interview. Medical records were available for all 655 participants (100%). Per person-year of follow-up over 12 months, those in the SPI group compared with those in the ESC group had 42% fewer suicide events (mean [SE], 1.82 [0.18] vs 3.11 [0.32]; mean [SE] difference, −1.30 [0.37]; *P* < .001), 55% fewer suicide attempts (mean [SE], 1.06 [0.14] vs 2.35 [0.33]; mean [SE] difference, −1.33 [0.38]; *P* < .001). Differences in weeks of suicidal ideation were not statistically significant (mean [SE], 10.39 [0.78] vs 12.86 [1.02]; mean [SE] difference, −2.47 [1.28]; *P* = .06). There were no other observed differences in outcomes.

**Conclusions and Relevance:**

In this randomized clinical trial of the SPI compared with ESC, those in the SPI group experienced reduced suicide risk by 42% in the year after jail release. These results suggest that SPI is effective for reducing suicide risk during this high-risk period.

**Trial Registration:**

ClinicalTrials.gov Identifier: NCT02759172

## Key Points

**Question:**

Does the Safety Planning Intervention (SPI) with telephone follow-up as an adjunct to enhanced standard care reduce suicide events 12 months after pretrial jail detention release?

**Findings:**

This randomized clinical trial of 800 individuals at risk for suicide who were recruited in jail, of whom 655 were released and followed up in the community, found that the SPI reduced the number of suicide events by 42% compared with enhanced standard care alone.

**Meaning:**

The findings of this study suggest that the SPI reduced suicide risk in the year after pretrial jail detention, a high-risk period in which 1 in 5 of all suicide deaths in the US occur.

## Teaser

This randomized clinical trial evaluates the effectiveness of the Safety Planning Intervention with telephone follow-up as an adjunct to enhanced standard care (ESC) compared with ESC alone for reducing suicide events in the 12 months following release from pretrial jail detention.

## Introduction

With more than 10 million admissions per year^1^ and short stays (often only a few days),^2^ US jails touch many individuals at high risk for suicide who are not well-connected with other service systems. Given that 1 in 5 US adults who die by suicide in the community have spent at least 1 night in jail in the year prior to their death,^3^ targeting suicide risk reduction as individuals leave jail detention could have a noticeable impact on national suicide rates.

This study reports results of the first randomized clinical trial, to our knowledge, of an intervention for suicide prevention in the high-risk year after jail release.^4^ The SPIRIT (Suicide Prevention Intervention for At-Risk Individuals in Transition) trial examined the effectiveness of the Safety Planning Intervention plus telephone follow-up (SPI) relative to enhanced standard care (ESC) for reduction of suicide events in the year after release from pretrial jail detention. SPI has a strong evidence base for suicide prevention in other crisis populations (eg, Veterans Affairs and civilian emergency departments, crisis lines),^5,6,7,8,9^ decreasing suicide behavior relative to treatment as usual by approximately 45% over 6 months.^5^

Study hypotheses were that over 12 months after jail release, participants randomized to the SPI group would have (1) fewer suicide events (a composite of suicide attempts and behaviors, suicide-related hospitalizations, and suicide deaths [primary outcome]); (2) fewer suicide attempts, weeks of active suicidal ideation (SI), severity of SI, psychiatric symptoms, and functioning (secondary outcome); and (3) longer time to first suicide event (secondary outcome). The study hypothesized that SPI would improve outcomes through increases in mental health treatment utilization, suicide-related problem-solving, and sense of belongingness.

## Methods

### Design and Setting

The methods for this 2-arm randomized clinical trial are described elsewhere,^4^ and the trial protocol is presented in Supplement 1. SPIRIT compared ESC with ESC plus the SPI among individuals in pretrial jail detention who reported past-month SI with intent and/or a suicide attempt. Participants were recruited from 2 midsize jails (Rhode Island Department of Corrections in Cranston and Genesee County Jail in Flint, Michigan) from May 11, 2016, to November 13, 2018. Assessments were conducted at baseline (in jail) and at 1, 4, 8, and 12 months’ postrelease intervals, including interviews and reviews of area hospital medical records. Follow-up assessments occurred by telephone, unless the participant was reincarcerated or in a controlled setting, and concluded on January 15, 2020, with health records received by August 4, 2021. Follow-up assessors were masked to condition assignment. Written informed consent was obtained privately by study staff.^4^ SPIRIT received approval from the Michigan State University institutional review board with oversight from the National Institute of Mental Health (NIMH) Data and Safety Monitoring Board-B. The trial followed the Consolidated Standards of Reporting Trials (CONSORT) reporting guideline.

### Participants

Eligible participants were unsentenced adults (aged 18 years or older) in pretrial detention with suicide risk, defined as a past-month attempt or intent (items 4 or 5 of the Columbia-Suicide Severity Rating Scale [C-SSRS] screener),^10,11^ with English fluency sufficient for verbal questionnaires. Exclusion criteria were an expected prison sentence without community release, no locator contacts, no telephone access, or inability to consent.^4^ Participants were recruited from mental health watch or through study announcements. Self-reported race and ethnicity categories included American Indian or Alaska Native, Black or African American, Hispanic, White, or all other racial groups (including Asian, Native Hawaiian or Other Pacific Islander, or other classifications nominated by the participants) or not specified. Race and ethnicity were assessed in the study to properly characterize the sample for external validity purposes.

### Randomization

Participants were randomly assigned 1:1 to the ESC or the ESC plus the SPI group by study staff after baseline assessment. Assignment was therefore masked. Assignment was stratified by jail site, sex, and lifetime suicide attempt history (yes or no). The study statistician (R.N.J.) prepared the randomizations schedule before recruitment started.

### Interventions

#### ESC

Standard jail care for individuals who present with suicidal ideation or behaviors involves in-jail assessment and stabilization, with little or no community follow-up.^4,12,13,14,15,16,17^ This study enhanced jail standard care with 12 months of postrelease monitoring and emergency referral.^4^

#### SPI

SPI, developed by Drs Stanley and Brown,^7^ is a brief focal suicide-prevention approach centered on collaborative development of a written safety plan. The safety plan is a prioritized written list—in the patient’s own words—of coping strategies and supports that individuals can use during or preceding suicidal crises. In this study, the SPI involved 1 in-jail session and a target of 4 (up to 8) postrelease telephone calls^8^ over 6 months with the same master’s degree–level community mental health center clinician for risk assessment, safety plan review, and service linkage.^4^ Clinicians were trained and supervised by study staff (L.M.W. and G.K.B.). Of 797 sessions, 33 (4.1%) were rated by study investigators (G.K.B. and Barbara Stanley, PhD [see acknowledgment in Additional Contributions]) using the SPI Rating Scale, version 2,^5^ yielding a mean (SD) fidelity score of 17.1 (3.1) out of 22, indicating satisfactory fidelity.

### Sample Size Calculation

The target population was individuals in pretrial jail detention returning to the community. Participants who went directly to prison or received long-term jail sentences were not followed up and were counted in attrition estimates.^4^ Because sentencing was independent of study condition, their exclusion did not affect internal validity. All participants initially released to the community were followed up for 12 months after release, regardless of reincarceration. Monte Carlo-based power estimates (Supplement 1) indicated 96.5% power to detect hypothesized main effects.^4^

### Measures

#### Suicide Events

Following the recommendations of Oquendo et al,^18^ the primary outcome, suicide events, was a composite count from baseline through 12 months after release from jail that included any of the following: suicide attempts (including deaths), suicide behaviors (eg, preparatory acts) per C-SSRS criteria,^10,11^ and suicide-related hospitalizations. Data were ascertained from interviews (C-SSRS,^10,11^ Treatment History Interview (THI)^19^), hospital records, and state or national registries. Participants provided release forms for medical record access across regional hospitals. Trained, masked staff used a structured protocol to code events based on discharge information and clinical notes. Suicide attempts were coded if indicated on hospital forms or clinical notes. A suicide attempt leading to hospitalization, as documented by medical records or self-report, was considered a single suicide event. Suicide deaths were coded when listed as such in death records. All data sources were reviewed for consistency.^4,20^

#### Suicide-Related Secondary Outcomes

Suicide attempts were a secondary outcome, measured as noted. Weeks of active SI were assessed using the Longitudinal Interval Follow-Up Evaluation (LIFE)^21^ calendar method, which provides weekly ratings.^4^ The LIFE was also used to assess time to first suicide event. SI severity was measured at baseline and at the worst point at each follow-up using the C-SSRS intensity subscale.^10^

#### Symptoms and Functioning

Psychiatric symptoms were assessed using the *Diagnostic and Statistical Manual of Mental Disorders* (Fifth Edition) (*DSM-5*) Cross-Cutting Symptom Measure.^22^ Functioning was measured using the Veterans RAND 12-Item Health Survey from the RAND Medical Outcomes Study.^23^ These measures were administered at all assessment time points.

#### Hypothesized Mechanisms of the Effects of the SPI

We defined treatment utilization (primary hypothesized mechanism) as the number of community outpatient mental health and substance use visits in the 3 months prior to baseline (for the base model) or since the last assessment (at follow-up, the putative mediator), as indexed by the THI. Suicide-related problem-solving (exploratory mechanism) was assessed using the Suicide-Related Coping Scale,^24^ a self-reported measure assessing knowledge and perceived self-efficacy using safety plan strategies to manage SI and urges. Belongingness (exploratory mechanism) was assessed using the Interpersonal Needs Questionnaire-12 belongingness subscale.^25,26^ Measures were assessed at all time points.

#### Sample Descriptors

Descriptive data included self-reported baseline demographics; diagnostic information (lifetime psychotic symptoms, a manic or hypomanic episode, and a major depressive episode) assessed using the Mini International Neuropsychiatric Interview, version 6.0.0 adapted for *DSM-5*,^27^ an interview with data extracted categorically; and posttraumatic stress disorder (PTSD) symptoms using the Life Events Checklist, in which scores range from 0 to 51, with higher scores reflecting more frequent exposure to traumatic events, and the PTSD Checklist for *DSM-5*,^28,29^ in which scores range from 0 to 80, with higher scores indicating greater severity of trauma-related distress. Details are provided in the trial protocol (Supplement 1) and baseline reports.^4,30^

### Statistical Analysis

Data analysis was completed from April 2023 to May 2025. The primary outcome (count of suicide events) was derived from all events detected in C-SSRS interviews, medical record reviews, suicide deaths, and suicide-related hospitalizations. Because medical record reviews covered all participants, no suicide event outcome data were missing. We used negative binomial regression to model this outcome due to its skewed distribution, adjusting for sex and baseline lifetime suicide events, and used an offset for available follow-up time. The model is summarized as predicted means and group differences. For participants who died during follow-up, counts were adjusted for truncated observation. Restricted cubic splines were used for control of baseline suicide events. Sensitivity analyses (eTable 3 in Supplement 2) tested models with Winsorization at the 95th percentile (baseline suicide events and outcomes) to explore potential outlier influence. Statistical significance was defined as 2-tailed *P* < .05.

Secondary outcomes were analyzed using regression models with follow-up outcomes predicted by baseline values, sex, and study condition. Cumulative suicide attempts used a negative binomial model. SI severity (C-SSRS), which included many 0s, was modeled with a repeated measures zero-inflated negative binomial model. Time to first suicidal event was analyzed using a proportional hazards model; proportionality was tested via Schoenfeld residuals. The *DSM-5* Cross-Cutting Symptom Measure (scores range from 0 to 92, with higher scores reflecting greater severity of psychiatric symptoms) and Veterans RAND 12-Item Health Survey (scores range from 0 to 100, with higher scores indicating greater functioning) outcomes were modeled using mixed-effects growth curves.

**Figure 1.**
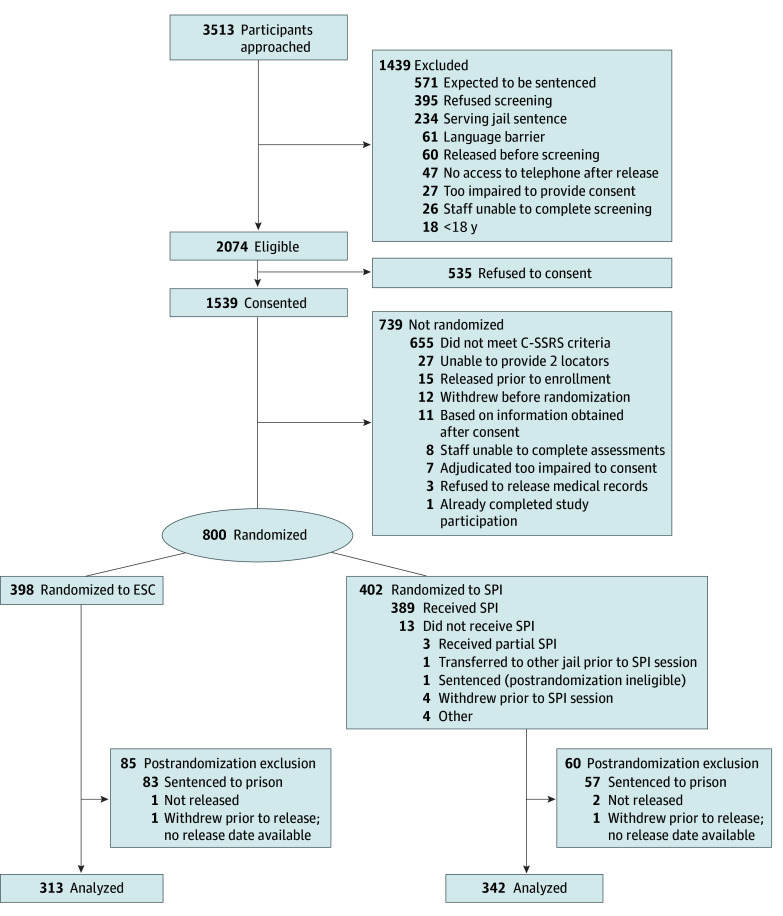
Participant Flow Diagram C-SSRS indicates Columbia-Suicide Severity Rating Scale; ESC, enhanced standard care; SPI, Safety Planning Intervention plus telephone follow-up.

Consistent with the intent-to-treat principle, analyses included all persons as randomized, although there was postrandomization eligibility (Figure 1) so our analysis can be considered a modified intent-to-treat analysis. Missing data were imputed using iterative chained equations.^30,31^ Missing data in the C-SSRS SI intensity model were addressed with maximum likelihood parameter estimation and were limited to participants with at least 1 C-SSRS follow-up.

To explore potential mediators—treatment utilization (number of outpatient mental health and substance use visits per the THI), suicide-related problem-solving (using the Suicide-Related Coping Scale), and belongingness (using the Interpersonal Needs Questionnaire-12)—we added each individually and jointly to the main outcome models. Mediation was evaluated by comparing the treatment effect estimate from the base model (*b*) with that from the model including the putative mediator (*b*′) and computing the percent change as 100 × (1 − *b*′)/*b*, where *b* is the treatment effect from the base model, and *b*′ is the effect after adjusting for the mediator. A 10% or more reduction in the treatment effect, per Maldonado and Greenland,^32^ indicated meaningful mediation.^33^ We also examined treatment group effects on each mediator (eTable 1 in Supplement 2).

Analyses were conducted with Stata, v.18.5 (Stata Corp LLC)^34^; R, v.4.4.3 (R Project for Statistical Computing)^35^; and Mplus, v.8.11 (Muthén & Muthén).^36^ All outcome models were prespecified and blinded to treatment assignment, and convergence issues were resolved prior to examining unblinded treatment effects. Consistent with an intent-to-treat framework, missing data were imputed using multiple imputations and the iterative chained equations approach^30,31^ or addressed with maximum likelihood parameter estimation methods.

## Results

### Participant Characteristics

Of the 800 participants randomized into the study (Figure 1), 655 (mean [SD] age, 33.0 [10.4] years; 182 females [28%] and 473 males [72%]) were released from jail and included in the outcome analyses. Among these participants, 342 were in the SPI group, and 313 were in the ESC group. In terms of race, 71 (11%) were American Indian or Alaska Native, 159 (24%) were Black or African American, 348 (52%) were White, and 77 (12%) were of other race group or were not specified. There were 87 Hispanic participants (13%) (Table 1).

**Table 1.** Participant Characteristics

Characteristic	Participants, No. (%)		
	Total (N = 655)	ESC group (n = 313)	SPI group (n = 342)
Age, mean (SD), y	33 (10.4)	33.2 (10.5)	32.8 (10.2)
Sex			
Female	182 (28)	84 (27)	98 (29)
Male	473 (72)	229 (73)	244 (71)
Race			
American Indian or Alaska Native	71 (11)	32 (10)	39 (11)
Black or African American	159 (24)	76 (24)	83 (24)
White	348 (53)	173 (55)	175 (51)
All other groups or not specified^a^	77 (12)	32 (10)	45 (13)
Hispanic ethnicity	87 (13)	38 (12)	49 (14)
Veteran status	38 (6)	17 (5)	21 (6)
MINI, lifetime^b^			
MDE	487 (74)	228 (73)	259 (76)
Manic or hypomanic episode	219 (33)	105 (34)	114 (33)
Psychosis symptoms	255 (39)	126 (40)	129 (38)
Life Events Checklist (experienced trauma)^c^	375 (57)	178 (57)	197 (58)
PTSD Checklist for *DSM-5*, mean (SD)^d^	34.2 (20.8)	32.7 (21.6)	35.6 (20.1)
Problematic substance use in past 3 mo (DUDIT or AUDIT >7)^b^	557 (85)	262 (84)	295 (86)
Suicide attempt in past 30 d	336 (51)	156 (50)	180 (53)
Lifetime history of any suicide attempt	586 (89)	280 (89)	306 (89)
Lifetime history of psychiatric hospitalization	470 (72)	225 (72)	245 (72)
Past year outpatient mental health treatment	479 (73)	222 (71)	257 (75)
Lifetime history of incarceration	617 (94)	295 (94)	322 (94)
Lifetime No. of arrests, median (IQR)	6 (2-14)	6 (2-14)	6 (3-14)
Baseline values of main outcomes			
Suicide events (lifetime, count), median (IQR)	9 (4-19)	9 (4-18)	8 (4-20)
Suicide attempts (lifetime, count), median (IQR)	4 (2-10)	4 (2-10)	4 (2-9)
Severity of suicidal ideation (lifetime, maximum), median (IQR)	20 (17-23)	21 (17-23)	20 (17-23)
*DSM-5* Cross-Cutting Symptom Measure, mean (SD)^e^	50.5 (16.2)	50.3 (16.0)	50.7 (16.3)
Functioning (VR-12), mean (SD)^f^	70.4 (16.1)	69.9 (16.6)	70.8 (15.7)

Abbreviations: AUDIT, alcohol use disorders identification test; *DSM-5*, *Diagnostic and Statistical Manual of Mental Disorders* (Fifth Edition); DUDIT, drug use disorders identification test; ESC, enhanced standard care; MDE, major depressive episode; MINI, Mini International Neuropsychiatric Interview; PTSD, posttraumatic stress disorder; SPI, Safety Planning Intervention plus telephone follow-up; VR-12, Veterans RAND 12-Item Health Survey.

^a^ Includes those identifying as Asian, Native Hawaiian or Other Pacific Islander, or other classifications nominated by the participants or not specified.

^b^ Presented as categorical data.

^c^ Scores range from 0 to 51, with higher scores indicating reflecting mor frequent exposure to traumatic events.

^d^ Total scores range from 0 to 80, with higher scores indicating greater severity of trauma-related distress.

^e^ Scores range from 0 to 92, with higher scores reflecting greater severity of psychiatric symptoms.

^f^ Scores range from 0 to 100, with higher scores reflecting greater functioning.

Arias et al^30^ provide detailed baseline characteristics of the sample. We note that among the 655 participants included in the follow-up sample, there were high rates of past 3-month substance use (557 [85%]), lifetime psychosis (255 [39%]), lifetime mania or hypomania (219 [33%]), and history of past psychiatric hospitalizations (470 [72%]) and prior suicide attempts (586 [89%]) (Table 1). Of these participants, 593 (91%) completed at least 1 follow-up interview. Review of medical records was completed for all 655 participants (100%). Unadjusted raw values for the primary and secondary outcomes at baseline and at the 12-month follow-up are provided in Table 2. We also report death data (which are counted in suicide event and attempt data) separately for descriptive purposes. Of the 16 deaths (2.4% of the follow-up sample) that occurred in the year after jail release, the manner of death was coded: suicide (3 [18.7%]), homicide (2 [12.5%]), natural cause (1 [6.3%]), and undetermined (10 [62.5%]). The cause of death in all 3 suicides was intentional overdose.

**Table 2.** Raw Main and Secondary Outcomes Observed Over 1 Year From Jail Release by Treatment Group

Outcome and time point	ESC group		SPI group	
	No.	Median (IQR)	No.	Median (IQR)
Suicide events	313	1 (0-3.0)	342	1 (0-2.0)
Count, No. (%)				
0	NA	146 (46.6)	NA	155 (45.3)
1	NA	59 (18.8)	NA	70 (20.5)
2-9	NA	81 (25.9)	NA	105 (30.7)
≥10	NA	27 (8.6)	NA	12 (3.5)
Suicide attempts	313	0 (0-1.0)	342	0 (0-1.0)
Count, No. (%)				
0	NA	194 (62.0)	NA	225 (65.8)
1	NA	44 (14.1)	NA	56 (16.4)
2-9	NA	58 (18.5)	NA	54 (15.8)
≥10	NA	17 (5.4)	NA	7 (2.0)
Time to first suicide event, wk	313	52 (7.5-52.0)	342	52 (10.3-52.0)
Count, No. (%)				
0	NA	49 (5.3)	NA	72 (7.6)
1-25	NA	192 (20.6)	NA	211 (22.4)
26-51	NA	34 (3.6)	NA	26 (2.8)
52	NA	7 (0.8)	NA	3 (0.3)
Severity of suicidal ideation (maximum over follow-up)	283	18 (14.0-21.0)	312	17 (12.0-21.0)
*DSM-5* Cross-Cutting Symptom Measure (maximum over follow-up)^a^	280	47 (30.5-60.0)	308	46 (30.0-57.8)
Functioning (VR-12 [minimum over follow-up])^b^	280	71.9 (58.5-87.4)	308	73.3 (58.1-85.1)

Abbreviations: *DSM-5*, *Diagnostic and Statistical Manual of Mental Disorders* (Fifth Edition); ESC, enhanced standard care; NA, not applicable; SPI, Safety Planning Intervention plus telephone follow-up; VR-12, Veterans RAND 12-Item Health Survey.

^a^ Scores range from 0 to 92, with higher scores reflecting greater severity of psychiatric symptoms.

^b^ Scores range from 0 to 100, with higher scores indicating greater functioning.

### Intervention Adherence

Among the 342 participants randomized to the SPI group and released from jail, 335 (98.0%) participated in the initial SPI session. Furthermore, 293 (85.7%) participated in at least 1 follow-up call, and 93 (27.2%) completed at least the targeted 4 or more follow-up calls during the 6 months after release (mean [SD], 2.36 [1.55]; range, 0-7).

### Primary Outcome

**Figure 2.**
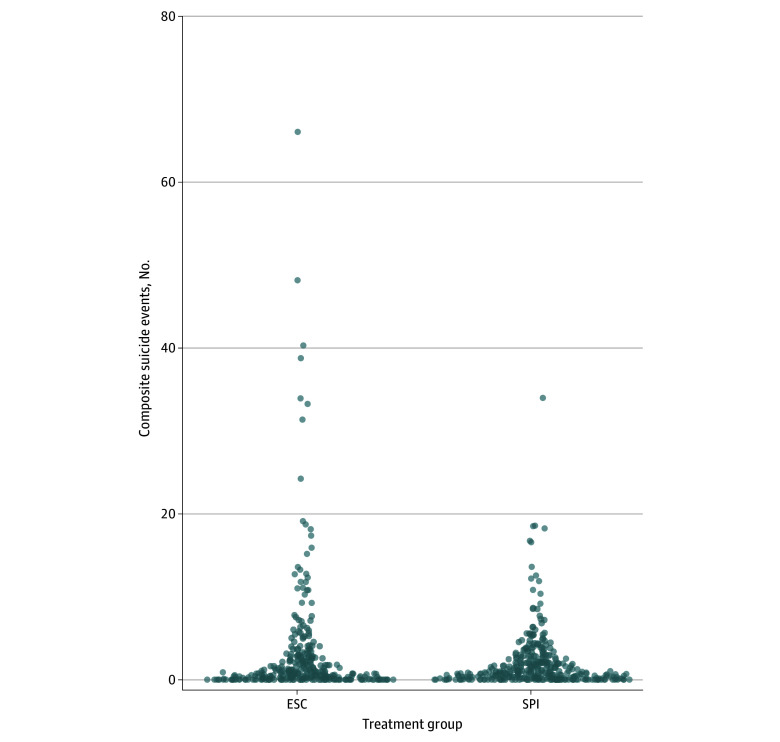
Count of Composite Suicide Attempts Over the Follow-Up Period by Treatment Group Distribution of composite suicide events (suicide attempts and behaviors, suicide-related hospitalizations, and suicide deaths) outcome. ESC indicates enhanced standard care; SPI, Safety Planning Intervention plus telephone follow-up.

Analyses showed fewer suicide events in the SPI group (mean [SE], 1.82 [0.18] per person-year of follow-up) relative to the ESC group (mean [SE], 3.11 [0.30] per person-year of follow-up) (Table 3). The net difference (mean [SE], −1.30 [0.37]) was statistically significant (*P* < .001) and is illustrated in Figure 2. The incidence rate ratio for SPI vs ESC was 0.58 (95% CI, 0.45-0.78), indicating a 42% lower rate of suicide events in the SPI group. Sensitivity analysis using Winsorization at the 95th percentile produced similar results (eTable 3 in Supplement 2).

**Table 3.** Main and Secondary Adjusted Outcomes Observed Less Than or Equal to 1 Year From Jail Release and Differences by Treatment Group

Outcome	Mean (SE)^a^		Difference	
	ESC group (n = 313)	SPI group (n = 342)	Mean (SE)^b^	*P* value
Suicide events	3.11 (0.32)	1.82 (0.18)	−1.30 (0.37)	<.001
Suicide attempts	2.35 (0.33)	1.06 (0.14)	−1.33 (0.38)	<.001
Duration of active suicidal ideation, wk^c^	12.86 (1.02)	10.39 (0.78)	−2.47 (1.28)	.06
Time to first suicide event or behavior, wk^d^	9.27 (2.40)	10.06 (2.56)	−0.78 (2.68)	.77
Severity of suicidal ideation (C-SSRS)	8.69 (0.51)	7.77 (0.48)	0.92 (0.58)	.11
*DSM-5* Cross-Cutting Symptom Measure^e^	29.57 (1.05)	28.60 (1.02)	−0.97 (1.15)	.40
Functioning (VR-12)^f^	85.05 (1.12)	83.35 (1.09)	−1.70 (1.56)	.28

Abbreviations: C-SSRS, Columbia-Suicide Severity Rating Scale; *DSM-5*, *Diagnostic and Statistical Manual of Mental Disorders* (Fifth Edition); ESC, enhanced standard care; SPI, Safety Planning Intervention plus telephone follow-up; VR-12, Veterans RAND 12-Item Health Survey.

^a^ Per person-year of follow-up.

^b^ Estimated means reflect adjustment for baseline value of the outcome variable (Table 1) or baseline composite suicide attempts, sex, and randomly assigned treatment group. Means reflect multiple imputation in the presence of missing data. SEs for weeks until first suicide event or behavior were obtained with the bootstrap.

^c^ Predicted over 52 weeks of follow-up.

^d^ Number of weeks until 25% of participants had first event or behavior.

^e^ Expected mean at 52 weeks. Scores range from 0 to 92, with higher scores reflecting greater severity of psychiatric symptoms.

^f^ Expected mean at 52 weeks. Scores range from 0 to 100, with higher scores indicating greater functioning.

### Secondary Outcomes

Analysis of secondary outcomes (Table 3) showed significantly fewer suicide attempts in the SPI group (mean [SE], 1.06 [0.14] per person-year of follow-up) vs the ESC group (mean [SE], 2.35 [0.33] per person-year of follow-up), and the mean (SE) difference was −1.33 (0.38) (*P* < .001). This corresponds to an incidence rate ratio of 0.45 (95% CI, 0.31-0.65), indicating a 55% lower rate of suicide attempts in the SPI group. Per person-year of follow-up, the SPI participants experienced a mean (SE) of 10.39 (0.78) weeks of active SI relative to a mean (SE) of 12.86 (1.02) weeks of active SI for participants in the ESC group, although this difference was not statistically significant (mean [SE] difference, −2.47 [1.28]; *P* = .06), with an incidence rate ratio of 0.81 (95% CI, 0.65-1.00). For time to first suicide event or behavior, the hazard ratio of the SPI relative to the ESC group was 0.94 (95% CI, 0.73-1.20), indicating no significant difference between groups. Other outcomes (SI severity, mental health symptoms, and functioning) were nonsignificant.

### Hypothesized Mechanisms

Results (eTable 1 and eFigure in Supplement 2) reveal no significant difference by condition for hypothesized mechanisms. The percentage of the treatment effect explained by each mediator is presented in eTable 2 in Supplement 2. Problem-solving explained 9.2% of the treatment effect in suicide events, and belongingness explained 5.3%, both falling below our 10% threshold. Adjusting for treatment utilization increased the magnitude of the estimated treatment effect by 1.9%, consistent with a suppression effect (ie, a variable that obscures the full effect of treatment rather than mediating it). For suicide attempts, none of the individual mediators met the 10% threshold. However, the combination of all 3 mediators explained approximately 10% of the treatment effect in both suicide events and attempts outcomes.

## Discussion

SPIRIT is the first randomized clinical trial, to our knowledge, of a suicide-prevention intervention in the year after release from pretrial jail detention, which is a high-risk period in which 1 in 5 of all US adult suicide decedents will die by suicide. Relative to the ESC group, those in the SPI group had fewer suicide events, suicide attempts, and weeks of active SI over the 12 months after release. There were no differences in other outcomes. Framing the clinical importance of these findings (Table 3), the SPI was associated with a relative risk reduction of 42% for suicide events and a 55% relative risk reduction for suicide attempts over the year after jail detention, with nearly 2.5 fewer weeks of active SI per person per year. Results also indicate that delivery to fidelity of the SPI is feasible within this setting and population, with 98% of those randomized to the SPI participating in the jail session and nearly 86% participating in at least 1 postrelease telephone session. Findings extend the general evidence base for SPI’s effectiveness^5,6,7,8,37^ for reducing suicide attempts and suicide events.

The findings are notable given the high-risk, multiply comorbid nature of the sample, including past 3-month substance use (85%), lifetime psychosis (39%), lifetime mania or hypomania (33%), and history of prior suicide attempts (89%) and psychiatric hospitalizations (72%) (Table 1). Per the previously published SPIRIT baseline study, one-third (34%) of those reporting prior suicide attempts made their first suicide attempt at or before age 10 years.^30^ Challenging life events and unstable life circumstances were pervasive.

In this study, SPI had focal effects on suicide events, suicide attempts, and ideation. Benefits of SPI did not appear to extend to overall mental health symptoms or functioning, consistent with SPI’s design as a focal suicide-prevention intervention.^7^ SPI is not designed to replace mental health or other interventions addressing social determinants of health such as housing or employment. We thus recommend SPI as an adjunct to other care,^37^ rather than as stand-alone treatment, when individuals returning to the community from jail have mental health, substance use, or other needs in addition to suicide prevention.

One clinical feature to keep in mind when using SPI in this population is that differences between SPI and ESC were driven by reducing numbers of attempts and events among people with many suicide attempts or events (Figure 2 and Table 3). SPI did not significantly reduce the likelihood of having 1 vs 0 events or attempts in the postjail year. This finding may reflect the sample’s high suicide risk and extensive histories of known risk factors like prior attempts, substance use, serious mental illness, and trauma.

### Next Steps

Next steps for this research include publishing cost-effectiveness and dose-response findings, moderators of treatment effects, pathways for implementation, and greater exploration of intervention mechanisms. In this study, SPI did not appear to impart its effects through a single mechanism that we tested but rather all together, possibly in concert with other mechanisms that could be the focus of future investigation. More pragmatically, the trial collected cost and cost-effectiveness data, which will be the focus of a subsequent planned report to inform efforts to implement and scale up the SPI for individuals at risk for suicide who pass through jail.

Having identified a great need (ie, 1 in 5 adult suicide decedents has spent a night in jail in the year prior to death) and an effective intervention (ie, SPI), efforts now focus on implementation. Jails can offer the SPI during detention but lack postrelease contact. Partnering with community clinicians for initial and follow-up sessions, as done in this study, may be most feasible. Another approach, being evaluated in the NIMH-funded National Center for Health and Justice Integration for Suicide Prevention,^38,39^ alerts community clinicians when their clients are released, to prompt check-in, other needed services, suicide risk assessment, and brief suicide interventions such as the SPI.

### Strengths and Limitations

This study has several strengths. These include a rigorous study design and assessment, internal validity (randomization, masked raters), external validity (a large, understudied sample; use of community clinicians), and strong follow-up rates (91% for interviews and 100% for medical records), with regular independent site monitoring and oversight from the NIMH Data and Safety Monitoring Board-B.

The study also has some limitations. The findings may not generalize to other settings or to individuals with less severe suicide risk. The study did not plan or use diagnostic assessment, precluding analysis of outcomes in the context of certain disorder presentations. By design, there was insufficient power to detect differences for suicide death. As is typical in suicide research,^40^ medical record data may have potentially underestimated suicide events.

## Conclusions

In this randomized clinical trial, SPI was found to be an effective intervention to reduce suicide events and attempts in the year after release from pretrial jail detention, which is a time in which a substantial proportion of all US suicides occur. We recommend use of SPI during and after jail incarceration for suicide prevention among those identified as at risk.

## Notes

Supplement 1. Trial Protocol

Supplement 2. **eTable 1.** Distribution of Hypothesized Mediators and Differences by Treatment Group **eTable 2.** Treatment Effect Mediation **eTable 3.** Sensitivity Analysis: Winsorized Cumulative Suicide Events and Cumulative Suicide Attempts Observed Over up to 12 Months After Jail Release, and Differences by Treatment Group, Adjusted **eFigure.** Distribution of Hypothesized Mediators Observed Over Follow-Up According to Treatment Group

Supplement 3.
Data Sharing Statement
