# Errors in Table 1

**DOI:** 10.1001/jamanetworkopen.2025.52190

**Published:** 2025-12-08

In the Original Investigation titled “Safety Planning vs Standard Care for Suicide Prevention After Pretrial Jail Detention: A Randomized Clinical Trial,”^1^ published November 10, 2025, there were errors in Table 1. The No. (%) of American Indian or Alaska Native participants should have been 32 (10) in the ESC group and 39 (11) in the SPI group; and the No. (%) of White participants should have been 173 (55) in the ESC group and 175 (51) in the SPI group. This article has been corrected.^1^
