# Supplementary material for: Safety Planning vs Standard Care for Suicide Prevention After Pretrial Jail Detention: A Randomized Clinical Trial
Source: JAMA Netw Open. 2025 Nov 10;8(11):e2543156. doi: 10.1001/jamanetworkopen.2025.43156 (PMC12603856; doi:10.1001/jamanetworkopen.2025.43156)
Supplement: Supplement 1. — Trial Protocol [file jamanetwopen-e2543156-s001.pdf]

CLINICAL STUDY PROTOCOL

SUICIDE RISK REDUCTION IN THE YEAR AFTER JAIL RELEASE

The SPIRIT Trial
(Suicide Prevention Intervention for at-Risk Individuals in Transition)

Version: 1
Version Date: 9-28-15

Principal Investigator (s):
Jennifer Johnson, Ph.D. & Lauren Weinstock, Ph.D.

Supported by:

The National Institute of Mental Health
The National Institute of Justice
NIH's Office of Behavioral and Social Science Research

U01 MH106660

Expanded Cover Page Information Sheet

Grant Number: U01 MH106660
Protocol Number 1

Principal Investigators [PIs] & Clinical Sites:

PI Name	Site #1	[redacted]	[redacted]	[redacted]
Jennifer Johnson, PhD	Michigan State University	[redacted]	[redacted]	[redacted]

PI Name	Site #2	[redacted]	[redacted]	[redacted]
Lauren Weinstock, PhD	Brown University	[redacted]	[redacted]	[redacted]

Study Phase and Target enrollment:
Phase of clinical investigation: Phase 3
Target enrollment: 800 Participants

NIMH Data and Safety Monitoring Board ☐ No ☒ Yes

Flesch-Kincaid reading level of consent form:
- NOTE: We ask all participants if they would like us to read the consent form aloud
- Flesch-Kincaid Grade Level = 7.5

Table of Contents

List of Abbreviations 6
Précis 7
Introduction/Background/Significance… 8
1A. Suicidality among jail detainees is a critical problem 8
1B. Critical need for targeted interventions for jail detainees 8
1C. Brief interventions are effective in other high risk populations 9
1D. Brief interventions have the potential for good uptake in jails 9
1E. Safety Planning Intervention is a brief, adjunctive intervention 9
1F. Mechanisms and fit for target population 10
1G. Innovation… 11
1H. Interventions 11
1I: Clinicians 12
2. Study Objectives 14
3. Subjects 15
3A. Description of Study Population 15
3B. Inclusion Criteria 15
3C. Exclusion Criteria 15
4. Study Design and Methods (including Consent Process) 16
4A. Study Overview 16
4B. Recruitment 17
4C. Screening and Consent Processes 18
4D. Study Procedures 19
4E. End of Participation 20
5. Storage of Data and Samples 21
5A. Sources of Materials 21
5B. End of the Study 21

5C. Plan for Rapid Sharing of Trial Data....21
6. Additional Considerations....22
7. Risks and Discomforts....23
7A. Sources of Materials....23
7B. Potential Risks....23
7C. Recruitment and Informed Consent....23
7D. Protection Against Risk....23
8. Subject Safety Monitoring....26
8A. Safety Monitoring Overview....26
8B. During Incarceration....26
8C. In the Community....27
8D. Withdrawal from the Study....28
8E. Criteria for Unblinding....28
9. Outcome Measures....29
10. Statistical Analysis....32
10A. Data Analysis....32
10B. Expected Attrition and Power Analysis....33
11. Human Subjects Involvement and Characteristics....35
11A. Subject Selection....35
11B. Jail Detainees as Human Subjects....35
11C. Sample Composition and Rationale....35
11D. Efforts to Achieve Targeted Sample Composition....35
11E. Safeguards for Vulnerable Populations…....36
11F. Qualifications of Investigators…....36
12. Anticipated Benefit....38
12A. Benefits of the Proposed Research to the Subjects and Others....38

13. Classification of Risk 39
13A. Naturalistic Risk of the Population Under Study..39
13B. Potential Risks Associated with Study Participation 39
13C. Overall Risk and Benefit Consideration...39
14. Consent Documents and Process 40
14A. Designation of Those Obtaining Consent..40
14B. Consent Procedures… 40
14C. Consent Documents… 40
15. Data and Safety Monitoring..41
15A. Data and Safety Monitor..41
15B. Data Monitoring Plan..41
15C. Site Monitoring Plan...42
15D. Safety Monitoring Plan 43
15E. Interim Analysis Plan..44
16. Alternative Therapies 45
17. Confidentiality 46
18. Conflict of Interest 47
19. Research and Travel Compensation 48
20. References 49
21. Attachments/Appendices..62

List of Abbreviations

- AE = adverse event
- AUC = area under the curve
- AUDIT = Alcohol Use Disorders Identification Test
- CD = compact disk
- CE = cost-effectiveness
- CEO = Chief Executive Officer
- CONSORT = CONsolidated Standards of Reporting Trials
- C-SSRS = Columbia Suicide Severity Rating Scale
- CTOBB = Clinical Trials Operations and Biostatistics Branch
- DAST-10 = Drug Abuse Screening Test-10
- DSM-5 = Diagnostic and Statistical Manual of Mental Disorders, 5th Edition
- ED = emergency department
- ED-SAFE = Emergency Department Safety and Follow-Up Evaluation
- [redacted]
- GCP = Good Clinical Practices
- HLM = Hierarchical Linear Modeling
- ICD = International Classification of Diseases
- INQ-12 = Interpersonal Needs Questionnaire-12
- IRB = Institutional Review Board
- LIFE = Longitudinal Interval Follow-up Evaluation
- [redacted]
- MINI = Mini International Neuropsychiatric Interview
- [redacted]
- NA = not applicable
- NIH = National Institutes of Health
- NIMH = National Institute of Mental Health
- PI = principal investigator
- QALY = quality adjusted life year
- RA = research assistant
- RCT = randomized controlled trial
- [redacted]
- [redacted]
- SAE = serious adverse event
- SAS = Statistical Analysis System
- SF-12 = Short Form-12
- SOC = Standard Care
- SPI = Safety Planning Intervention
- SPSS = Statistical Package for the Social Sciences
- SRM = Site Review Meeting
- THI = Treatment History Interview
- UP = unanticipated problem
- U.S. = United States

Précis

Objective: This is a randomized controlled trial (RCT) in 2 jail systems to evaluate the effectiveness and cost-effectiveness of Stanley and Brown's Safety Planning Intervention (SPI) to reduce suicide events (attempts, suicide behaviors, suicide-related hospitalizations, and suicide deaths) in the year following jail release.

Study population: The sample will include 800 (male and female) pretrial jail detainees who are at risk for suicide events (i.e., they endorse suicidal ideation with some intent to act or a suicide attempt in the past month). The sample will be recruited from two jails: [redacted].

Design: This study is a randomized controlled trial to evaluate the effectiveness and cost-effectiveness of SPI to reduce suicide events (attempts, suicide behaviors, suicide-related hospitalizations, and suicide deaths) in the year following jail release. We recruit and follow participants from two jail systems ([redacted]).

- SPI will consist of safety planning during jail detention coupled with post-release follow-up phone calls to review the safety plan and problem-solve barriers to use of safety behaviors after jail release. SPI is provided by clinicians from the community mental health centers providing standard, existing re-entry services.
- The control condition will be Standard Care (SOC). The current *standard* strategy for caring for suicidal jail detainees is assessment and psychiatric stabilization while in jail with essentially no community follow-up; we will provide post-release monitoring and emergency referral in keeping with ethical obligations to trial participants.
- Assessments will occur at baseline, and 1, 4, 8, and 12 months post-release.

Outcomes include:

1. Number of suicide events (a composite of attempts, behaviors, suicide-related hospitalizations, and suicide deaths) in the year following jail release (*primary*)
2. Number of suicide attempts, weeks of active suicidal ideation, severity of suicide ideation, time to first suicide event, psychiatric symptoms, and functioning *(secondary)*
3. We hypothesize that SPI will increase (a) treatment utilization, (b) suicide-related problem-solving, and (c) sense of belongingness, which will serve as mechanisms of SPI's effect on suicide events.
4. Cost, cost-offsets, and cost-effectiveness (which drive adoption and sustainability in re-entry settings)

1. Introduction/Background/Significance

1A. Suicidality among jail detainees is a critical problem of public health significance.

There were nearly twelve million admissions to U.S. jails in 2012.[1] In fact, the U.S. has the highest incarceration rate in the world.[2,3] Individuals who become incarcerated face numerous health disparities. Rates of past-year mental health (56%) and substance use (66%) disorders,[4,5-13] HIV,[7,14,15] hepatitis,[7] and tuberculosis[7] are dramatically elevated among incarcerated individuals, 68% of whom are from racial or ethnic minority groups[16] and 70% of whom had less than $2,000 of personal income in the month before arrest.[17] Around half (40-50%) of incarcerated individuals report lifetime suicidal ideation or behavior and 13-20% have attempted suicide.[18-20] Incarcerated individuals die by suicide at a rate that is 8 to 14 times greater than the general population.[21-23]

Unlike prison, where individuals have been sentenced and typically stay from months to years, jail detainees are either pretrial (unsentenced) and may be released on bond, or are serving very short sentences. The weekly turnover rate in U.S. jails is 65%.[24] Most people who are arrested[25] are booked into jails (12 million ad-missions in the U.S. in 2012).[26] Only a few are subsequently sentenced to prison (~700,000 admissions per year).[27] The majority of people passing through jails are charged with misdemeanors, such as public drunken-ness, trespassing, shoplifting, and public disturbances.[28] In prisons, longer stays offer opportunities to receive treatment and post-release treatment planning. In contrast, most individuals are in jail *only a few days* and release times are unpredictable, meaning that intensive, long-term treatment is usually not possible and post-release treatment planning occurs quickly if at all.[28-30] Brief, flexible interventions are needed for jail settings.

1B. There is a critical need for targeted suicide prevention interventions for jail detainees, especially after jail release.

Arrest and jail detention represents an acute stressor that exacerbates suicide risk in an already high risk, vulnerable population. In contrast to suicide rates of 12 per 100,000 in the general population and 10-14 per 100,000 in state and federal prisons, the rate of suicide <u>during</u> jail detention is roughly 35 per 100,000.[31,32] Although not the subject of formal randomized intervention trials, increased awareness, quality improvement, and implementation of clinical guidelines for screening and safety during jail detention (e.g., Shield of Care for incarcerated teens,[33] publications by the US Marshals[34] and others[35]) have been credited for reducing the *<u>in-jail</u>* suicide death rate from over 100 per 100,000 twenty years ago.[31,32,36]

Less attention has been paid to reducing suicide risk and mortality following release to the community,[37-40] when individuals have increased access to lethal means (substances, firearms, vehicles) and are faced with numerous financial, legal, and social stressors.[35,41-45] The resurgence of problems, including substance use,[46] risky behavior,[47,48] victimization,[43,48,49] and re-arrest[50] during this period is typical. At least one study showed suicide to be the leading cause of mortality following release from jail,[39] with evidence that suicide accounted for 20% of all deaths during this period. Others have revealed suicide to be among the top 3 causes of post-release mortality, along with accidental drug overdose and homicide.[39,40,51-53] A recent meta-analysis found the average rates of death by suicide in the first post-release year to be 128 per 100,000 (range: 41-204 per 100,000), meaning that more people die by suicide in the year *after release* from incarceration than during jail detention.[54,55] Reducing suicide risk in the year after jail detention could have a noticeable impact on national suicide rates,[56] given that National Violent Death Reporting System general population data indicate that roughly 10% of *<u>all suicides</u>* with known circumstances occur in the context of a recent criminal legal stressor (typically arrest and jail detention). If the effects of brief suicide prevention interventions found in other at-risk populations (relative risks of 1.6-2.6[57-61] for attempts and 11.0 for suicide deaths[62]) hold for recently re-leased jail detainees, implementation of this intervention could result in a 5%-9% reduction in all U.S. suicides.

In sum, jail detention is a marker for increased suicide risk: jail detainees are a high-risk, low-resource population with complex psychiatric, health, housing, and employment

challenges,[63] who are facing a major life stressor (i.e., arrest). Release to the community decreases supervision and increases access to lethal means. Lack of education, poverty, victimization, homelessness, isolation, and poor employment skills complicate care and increase morbidity and mortality.[64-71] Suicide intervention research for this population is lacking: *this study will be the first RCT of any intervention for suicide risk reduction following release from jail*.

1C. Brief interventions are effective at reducing suicide risk in other high risk populations.

Although jail detention affords a critical moment of opportunity to deliver suicide prevention intervention to a vulnerable population, individuals are typically detained for only a few days (median length of stay = 4 days [redacted]), requiring rapid, flexible, and implementable intervention. Fortunately, there is an emerging evidence base supporting the effectiveness of brief suicide prevention interventions,[72] typically consisting of one in-person session and then telephone or mail follow-up,[73] in other high risk populations. For example, among large samples of suicidal emergency department (ED) patients, brief intervention and telephone contact has been shown to decrease subsequent rates of suicide attempt, especially in the first month following the index ED visit,[74] and to decrease subsequent suicide deaths (0.2% vs. 2.2%) and overall mortality by any cause (1.3% vs. 2.7%) relative to treatment as usual.[75] Randomized trials have also found low intensity contact letter interventions (i.e., mailing brief, caring outreach messages) to yield reduced cumulative number of repeat suicide attempts, suicide events (i.e., attempts, hospitalizations), and suicide deaths among recently hospitalized patients.[76,77-79] Other brief interventions, such as the Collaborative Assessment and Management of Suicidality (CAMS)[80] and Applied Suicide Intervention Skills Training (ASIST)[81] have also shown promise.

1D. Brief suicide interventions have the potential for good uptake in jails.

Although not meant to replace other more intensive treatment (e.g., Dialectical Behavior Therapy[82]), the fact that these brief interventions were developed for delivery in transitory, crisis-oriented settings such as EDs and inpatient units make them ideal for delivery in jail (another transitory, crisis-oriented setting). Brief, low intensity interventions that can be delivered by a broad range of clinicians are also more feasible and potentially scalable within the complex, resource-challenged criminal justice and affiliated community mental health contexts.[12,83-91] Stanley and Brown's Safety Planning intervention (SPI; see 1E) has excellent potential for uptake and sustainability in these settings. SPI is brief, flexible, and collaborative, important characteristics for jail re-entry interventions. SPI is very structured and can be delivered by a broad range of clinicians. SPI is scalable with existing service structures and the typical clinicians that work within them (see 1I).

1E. Stanley and Brown's Safety Planning Intervention (SPI)[92] is a brief, adjunctive intervention designed to reduce subsequent suicidal behavior in high-risk populations.[92-94]

SPI has been identified as a 'Best Practice' in the joint Suicide Prevention Resource Center-American Foundation for Suicide Prevention (SPRC-AFSP) Registry. The core element of SPI is the collaborative development of the Safety Plan, which is a prioritized written list – in the patient's own words – of coping strategies and supports that individuals can use during or preceding suicidal crises. To address challenges of continuity of care across vulnerable transitions (e.g., from ED to community treatment, from inpatient to outpatient treatment), SPI often includes telephone follow-up with the same treatment provider to conduct periodic risk assessment and mood checks, review the Safety Plan, problem-solve obstacles to treatment, and assist with linkage to services.[92,93,95] SPI incorporates evidence-based suicide prevention strategies, including facilitation of suicide-related safety skills,[96,97] identification of social supports and emergency contacts,[98-101] lethal means restriction,[102-106] service linkage,[107] and motivational enhancement[108-110] to promote community treatment engagement. An emerging evidence base supports SPI for suicide prevention. Recent data support the acceptability and

effectiveness of SPI for individuals at risk for suicide across a number of acute settings (e.g., VA and civilian EDs, National Suicide Prevention Lifeline, NY State Office of Mental Health)[92,93,111,112] for the reduction of suicidal ideation and behaviors among high-risk patient groups.[93,111,113-115] As in these urgent care settings, the goal with SPI in jail settings is not to solve all of detainees' challenges with a single brief intervention, but rather to intervene in targeted ways to reduce suicide risk and to improve linkage to mental health care and other needed services.

1F. Mechanisms and fit for target population.

We propose that (a) treatment utilization, (b) suicide-related problem-solving, and (c) belongingness will serve as mechanisms for SPI's effects on suicide events.

Treatment utilization (primary) is strongly linked to suicide risk reduction.[116-118] SPI increases treatment engagement (see 1H). SPI helps problem-solve service linkage issues, which present huge challenges for re-entering individuals given difficulties with transportation, service availability, stigma, and trust of medical institutions.[44,49,119-121] In fact, service linkage is recognized by jails as the primary, top-priority barrier to post-release health outcomes.[19,122-128] SPI also works to increase motivation for service engagement.[129-131] Finally, continuity of at least one provider across the transition from jail to the community has been described as essential for post-release care.[48,49,132,133] SPI's blended in-person/phone approach delivered by community mental health center clinicians (see 1I) will provide this continuity, responding to recommendations of the National Confidential Inquiry into Suicide and Homicide[56] to reduce community-level suicide rates through better communication and cooperation between justice and community mental health agencies.

Suicide-related problem solving (exploratory). There is a robust association between problem solving deficits, which are prevalent among incarcerated populations,[20,134-136] and suicide risk in community[137-139] and incarcerated[20,134,140] populations. Stressful life events, also common in our target population,[35,41-45] can also interfere with cognitive processes needed for deliberation, further priming poor and impulsive decision making.[141-143] SPI facilitates the use of safety-related coping skills (skills which reduce suicide risk[96,97]) for managing crises using a template for rehearsing safety behaviors. The written safety plan, developed when participants are in controlled setting with time to deliberate (i.e., jail), allows individuals the opportunity to make decisions that support safety in future situations when their ability to generate and weigh options might be more limited (e.g., in the context of an acute life stressor, psychiatric symptom exacerbation, substance use, fatigue, etc.) or when the environment is less controlled (i.e., less supervision, more access to lethal means). Thus, SPI helps at-risk individuals make and enact safety decisions so that they do not need to generate, weigh, and execute options for the first time when faced with an acute crisis and resulting reduced capacity to do so.[141-143] These safety habits and structures put into place during incarceration safeguard against future difficult moments.

Belongingness (exploratory). Emerging from the Interpersonal-Psychological Theory of Suicide,[144] there is evidence that a sense of thwarted belongingness, defined as the belief that one does not have meaningful relationships with others or that others cannot relate to an individual's experience, is associated with increased suicide risk.[145] This construct of thwarted belongingness is especially relevant to criminal justice-involved populations because they are often socially marginalized. In fact, loneliness, interpersonal conflicts or stress, and having no one with whom to discuss bad news are strong predictors of suicide attempts and deaths in incarcerated samples.[35,146-149] SPI harnesses social supports and identifies contacts to reduce isolation in times of crisis, enhancing belongingness. Moreover, because recent detainees are often disenfranchised and marginalized,[49] receiving outreach in the form of caring telephone calls may also serve to increase a sense of belongingness.[132,150] Our previous research has shown post-release telephone outreach to be meaningful and powerful among re-entering individuals.[133,150-152] Overall, SPI is well matched to both the target population *and* target systems for ultimate dissemination and implementation. *However, there is no previous large-scale test of this intervention (or any other) for reducing suicidality following release from jail*

detention.

1G. Innovation: This study will be the first RCT of any intervention to reduce suicide risk after jail detention.

This is high-risk period, and a high-risk, large, and virtually unstudied population. NIMH's 2014 Research Agenda for Suicide Prevention[153] prioritizes intervention research in settings that are catchment areas for at-risk individuals; it *specifically mentions jail* as one of these areas. Given that there are no existing research-supported approaches, this trial addresses the compelling unmet need for effective interventions to reduce suicide risk after jail release and addresses an important gap in the literature. If shown effective, SPI has the potential to change clinical practice (see 1C, 1I) and measurably reduce U.S. suicide rates (see 1B).

1H. Interventions

SPI will include one in-person meeting in jail to create a safety plan and then 4 telephone meetings after release to review the safety plan and address barriers to implementing safety behaviors (see Appendix A). Post-release telephone contacts maximize feasibility given post-release transportation challenges, and reflect preferences of re-entering individuals. Furthermore, our research with previously incarcerated individuals as well as with suicidal individuals in the community has indicated that telephone follow-up intervention is feasible, acceptable, and powerful in building trust and reducing risk among these disenfranchised, often isolated, populations (see 1H). Telephone contacts are a cost-effective procedure[154] for contacting patients and have been found to be effective in a number of disorders,[155-172] including depression.[157] We have extensive experience in providing phone interventions.[173-177]

The *Initial Session During Jail Detention* will take place in person at the jail and will include a comprehensive clinical suicide risk assessment and development of a Safety Plan, a prioritized list of coping strategies and sources of support that patients can use during or preceding suicidal crises. The Safety Plan uses a simple, easy-to-follow format meant to enhance individuals' sense of self-control over suicidal urges and thoughts. During the risk assessment, the clinician obtains an accurate account of the events that transpired before, during, and after the most recent suicidal crisis. This description may include the activating events as well as the patient's reactions to them. This discussion helps to facilitate the identification of warning signs to be included on the Safety Plan, as well as the identification of specific strategies or behaviors that may have been used to alleviate the crisis. The SPI hierarchically-arranged steps are: (1) Identification of warning signs; (2) Use of internal coping strategies including distraction; (3) Social contact with others who may offer support and distraction from the crisis, without discussing suicidal thoughts; (4) Contacting family members or friends who may help resolve a crisis and with whom suicidality can be discussed; (5) Professional contacts including crisis hotline number, nearest ED address, clinicians' contact; (6) Restriction of access to lethal means. Patients are instructed to first recognize when they are in or at risk for crisis (Step 1) and then to follow Steps 2 through 6 as outlined in the plan. If following the instructions outlined in Step 2 fails to decrease the level of suicide risk, then the next step is followed, and so forth. SPI conveys a very clear path to follow. Since people cannot think clearly during emergencies, a clear predetermined strategy is most effective to mitigate risk.[96,97]

Post-Release Telephone Sessions. The same clinician who met with the individual in jail will contact him or her 4 times by phone at key time points (within the first week, 1 month, 3 months, and 6 months) after jail release, providing the most frequent contact in the highest risk period just after release. For individuals in crisis, clinicians have the option of scheduling an additional 2 calls. Calls are structured and have an agenda: (1) mood check and suicide risk assessment; (2) review and revise the safety plan; and (3) review treatment options and problem-solve obstacles to treatment. Clinicians ask when the person's next mental health appointment is scheduled, assess motivational and structural barriers to attendance, and help address these barriers. Clinicians can help identify treatment and other resources and facilitate appointments for patients if needed. If patients are assessed to be at acute risk, we will take appropriate action to maintain their safety, which may include contacting existing providers, ED referral, or

calling the police (see Human Subjects).

Standard Care. As is the case in most jails nationally,[23,34,35,178-180] Standard Care (SOC) for pretrial jail detainees at the study sites ([redacted]) is screening (by an intake worker) and assessment of risk (by a social worker). Individuals considered to be at acute risk of suicide are placed on psychiatric observation in the jail, where they are stabilized to the extent possible during their jail detention (i.e., they may be high, manic, or floridly psychotic when detained and may only be in jail for a few days, often coming in on a Friday night and being released from court Monday). If jail staff determine a detainee's imminent suicide risk to decrease while they are in jail, they leave observation, enter the general jail population, and then are released with no community follow-up. If an individual on observation is released on bail, the jail will ask the person picking the detainee up to take him or her to the ED for evaluation; no further follow-up is provided. If an individual on observation goes to court, the jail provides a letter asking the court to have the person evaluated by a mental health professional before releasing him or her; no further follow-up is provided. Individuals identified by the jail as having a severe mental illness (i.e., schizophrenia, bipolar disorder) are provided with post-release appointments. Thus, research assessment and emergency referral for trial participants on the basis of suicidality should be considered "enhanced" care compared to current jail practice, in keeping with ethical obligations. We will also provide handouts with an overview of local community treatment and other relevant community resources (e.g., housing, food) to all study participants.

1I: Clinicians

We will hire the community clinicians who would eventually deliver this intervention in regular practice to moonlight as clinicians on this study. They will be recruited from the agencies contracted to provide mental health services to individuals re-entering their respective communities from jail and prison. In [redacted], the [redacted] is the community mental health center serving the area to which the largest number of re-entering individuals in [redacted] returns. The [redacted] director of justice-related treatment services, [redacted], is a Consultant on this proposal. In [redacted], [redacted] serves re-entering individuals; its CEO, [redacted], is also a Consultant. Because these agencies' clinicians serve a large number of re-entering individuals, they are experienced in working with justice-involved clients and with common co-occurring problems, such as substance use and partner violence. [redacted] have well-established procedures for hiring, training, supervising, retaining, and replacing (when they change employment) these community clinicians in their previous in-person and phone-based and prison mental health and suicide prevention intervention studies.

We will hire and train an initial cohort of 16 master's-level clinicians (to cover the two jails 7 days per week plus back-ups) from the [redacted] and [redacted] to moonlight on this study. Because of high rates of turnover among community mental health clinicians, we anticipate training 24-28 over the 2.75-year intervention period. [redacted] will lead the training. Training will use the program developed by [redacted] and used successfully to train clinicians to fidelity within their previous study. This day-long, in-person initial training consists of reviewing the SPI rationale, materials, and strategies; audio-taped demonstrations; and live practice sessions with feedback. [redacted] will serve as the primary clinical supervisor for this study, with help from [redacted] and input from [redacted].

As in our previous and ongoing studies, in-jail treatment sessions are recorded using credit-card sized digital audio recorders that we are able to bring in and out of the jail. Recording of phone sessions uses a digital audio recorder connected to a telephone headset system and transmitter patch. As in previous and ongoing studies, study clinicians upload the recordings to our secure research audio/video server from their (remote) computers. Study supervisors, consultants, and fidelity raters can then listen to study intervention sessions from their (local or remote) computers, and supervision takes place by phone. [redacted] currently uses this system to supervise prison research clinicians in 2 states. Supervision will include weekly review of clinicians' audio-taped sessions, weekly group supervision and case discussion by

phone, and individual phone consultation on an as-needed basis. With help from [redacted], we will offer at least yearly 'booster' in-person training sessions to train new clinicians and provide review training for continuing study clinicians. Fidelity ratings will occur throughout the RCT; retraining will take place as necessary.

2. Study Objectives

This RCT evaluates the effectiveness and cost-effectiveness of SPI for reducing suicide events (attempts, suicide behaviors, and suicide-related hospitalizations and emergency department visits) among 800 suicidal pretrial jail detainees from two jails in the year following jail release. This study will be the first randomized evaluation of a suicide prevention intervention in the vulnerable year after jail release. Outcomes include:

1. Number of suicide events (a composite of attempts, behaviors, suicide-related hospitalizations, and suicide deaths) in the year following jail release (*primary*)
2. Number of suicide attempts, weeks of active suicidal ideation, severity of suicide ideation, time to first suicide event, psychiatric symptoms, and functioning *(secondary)*
3. We hypothesize that SPI will increase (a) treatment utilization, (b) suicide-related problem-solving, and (c) sense of belongingness, which will serve as mechanisms of SPI's effect on suicide events.
4. Cost, cost-offsets, and cost-effectiveness (which drive adoption and sustainability in re-entry settings)

3. Subjects

3A. Description of Study Population

Our target population is suicidal pretrial jail detainees who are returning to the community. We will exclude individuals who expect to be sentenced to prison. However, we expect 6-8% of the pretrial jail detainee participants we consent who do *not* expect to be sentenced to prison will be sentenced anyway. These individuals will not leave jail for the community (i.e., will go directly to prison, not home), meaning that they are not eligible for this study (suicide prevention in the year after release from pretrial jail detention). Therefore, individuals who go to prison directly from jail rather than back to the community will not be followed, and have been included in our study attrition estimates. This is a standard approach taken in other re-entry studies (e.g., R01 AA021732; U01DA016191[13]) that must consent participants when their sentencing or release status is still unknown. Sentencing will occur independent of study condition, so their exclusion from analysis (no "at-risk" community months) will be unlikely to influence internal validity. We will follow all remaining participants who are released from jail to the community after the index incarceration through the 12-month post-release period regardless of reincarceration, continued participation in SPI or SOC, or subsequent suicide attempts or hospitalizations. Of the 92-94% of participants who are released from jail to the community after the index incarceration, we conservatively estimate that post-release follow-up rates will be 82% at 1 month, 80% at 4 months, 75% at 8 months, and 70% at 12 months, with 85% of participants providing data for at least one post-release follow-up interview. Post-release follow-up rates in [redacted] previous and ongoing studies have been higher than this. Therefore, we expect that 78% (85% of the 92% who are released from jail) of the 800 enrolled participants will provide evaluable follow-up data. Count (e.g., suicide events) data from missed follow-ups will be gathered at later follow-ups when they occur, and we will collect medical record and death record data on all eligible (i.e., released) participants. Study dropouts will not be replaced; attrition has been accounted for in power and sample size estimates.

3B. Inclusion Criteria

Unsentenced male and female pretrial jail detainees will be eligible for the study if they are: (1) 18+ years of age; (2) at risk for suicide, operationalized as a response of "yes" on item 4 or greater on the initial 5 Columbia Suicide Severity Rating Scale (C-SSRS) screening questions, indicating the presence of at least some active suicide ideation with some intent to act in the past month (i.e., individuals at higher risk, such as those who report intent with specific plan and/or suicide attempt/s in the last month, will also be included); and (3) speak and understand English well enough to understand questionnaires when they are read aloud (~98%).

3C. Exclusion Criteria

We will exclude people who: (1) expect to be sentenced to prison (i.e., expect to go directly to prison, not home, from the jail), (2) cannot provide the name and contact information of at least two locator persons (~6%), and/or (3) do not have access to any telephone. In our previous jail studies, most people screened (92%) *owned* a phone and virtually all had access to a phone through owning one, a relative/friend, or an agency. We regularly contacted participants by phone.

4. Study Design and Methods (including Consent Process)

4A. Study Overview

This study is a randomized controlled trial to evaluate the effectiveness and cost-effectiveness of Stanley and Brown's Safety Planning Intervention (SPI to reduce) suicide events (attempts, suicide behaviors, suicide-related hospitalizations, and suicide deaths) in the year following jail release. We will recruit and follow 800 participants from two jails: [redacted]

Study assessments take place at baseline, and then 1, 4, 8, and 12 months post-release. Baseline assessments occur at the jail. Post-release assessments take place by telephone, unless the person has been reincarcerated. If the person is reincarcerated, affected study assessments will take place in person at the correctional facility. Participants are enrolled in the study until either:

1. The 12-month post-release assessment has been completed or deemed "missed."
2. They are determined to be ineligible. For example, some participants may sign consent before completing assessment for full eligibility. Furthermore, if someone who has consented is sentenced to jail or prison time without being released to the community following pretrial jail detention, they are not eligible for the study and will not be followed (see Section 10 section on Attrition). Participants who are released to the community following study enrollment and then are subsequently reincarcerated are eligible and will still be followed.

The participants who are randomized to the SPI condition will also receive SPI contacts. This consists of an in-person meeting with a study counselor at the jail, and then 2-6 follow-up phone calls with the counselor in the 6 months after jail release.

Randomization and blinding. Randomization to SPI or SOC in a 1:1 ratio will occur in the jail after the baseline assessment; therefore, all baseline assessments will be "blind." Randomization will be stratified by jail, gender, and history of suicide attempts (yes or no). As in [redacted] ongoing jail study, study counselors (who are available every day of recruitment; see Budget Justification) will meet with those assigned to the intervention condition within 24 hours of randomization. Typically, we recruit participants in the morning to meet with the interventionist/s scheduled to come to the jail that afternoon or evening. Immediately after randomization, RAs will also review the study follow-up schedule, means of contacting the research staff, and participants' contacts with all participants. A different research assistant, who is blind to intervention assignment, will perform telephone follow-up assessments. Information about a participants' experimental condition will be kept separately from the databases where research assistants will enter assessment data. Patients will be instructed at each visit that they are not to make any mention of their treatment condition while they are being interviewed by their assigned rater. At the beginning of each rating session, the rater will remind the patient that she/he is not to mention treatment condition during the interview with the rater.

The study statistician, [redacted], will prepare the randomization schedule before the first participant is enrolled.

Proposed Staffing

Role	Blind to Tx Condition	Access to Study Data
Principal Investigators	No	Limited to demographics, randomization, and blinded outcome data
Project coordinators	No	Yes
Study clinicians (community clinicians moonlighting for the study)	No	No
Clinical supervisors	No	No
Research assistants (RAs)	Depends*	Only for data entry

**The RA that does a particular participant's intake assessment will NOT be blinded for that participant. The unblinded intake RA will help schedule follow-up assessments, but a different RA (who is blinded for that participant) will conduct the follow-up assessments. Therefore, each RA will be blinded to about 83% of participants, and will conduct follow-up assessments for only those participants.*

4B. Recruitment

Participants (pretrial jail detainees) will be recruited from the [redacted] and from the [redacted].

- As part of their routine practices, the jails in each system [redacted] screen each detainee for the presence of SI at the time of intake. This screening has two levels; the first involves screening by non-clinicians: in intake officer in one jail and a police officer in the other jail. This first level of screening includes prescribed yes/no questions. Those who screen positive at this level are put on suicide watch until they can be evaluated by a jail mental health clinician (typically a licensed social worker or psychiatrist). Depending on what day of the week and what time of day they are admitted (e.g., Friday night), it may be a couple of days before the clinician can see them. The licensed clinician then interviews them and has the choice to clear them into the general population or maintain them on suicide watch. Data from our two jail study sites indicate that approximately 7-10% of detainees in our systems will be placed on suicide precautions at some point during their jail detention.
- Our recruitment strategy is to begin study screening with those on suicide precaution. This is easy because these individuals are typically housed in a single wing at each jail. Given that some individuals with suicidality may have been cleared into general population already, or (rarely) may not have been identified by the jail's initial screening, if we still have time on a given day after enrolling eligible individuals on suicide watch, we will supplement recruitment by approaching detainees within the general population to assess their interest in study participation. To do so, we will distribute "slips" of paper briefly describing the study to all detainees on a given wing; detainees can indicate interest or lack of interest in meeting with the study RA on the slips, which are discreetly returned to the RA (see Human Subject section). The RA then approaches each detainee privately to discuss study participation further, and to begin the informed consent process if there is an interest in participation. This is an approach we have used successfully in many of [redacted] previous jail and prison studies.

Anticipated accrual rate. We will enroll an average of 30 participants (who meet study criteria and consent to participate) per month for 27 months, resulting in an enrolled sample size of 800

(~500 in [redacted] ~300 in [redacted]).

- However, if for some unforeseen reason recruitment lags, [redacted] has ongoing research in [redacted] jails, which house a population 5-6 times that of [redacted], and [redacted] has ongoing research in jails in [redacted]. We could expand this study to those locations if needed.
- Enrolling 25%, 50%, 75%, and 100% of the sample (Months 4-30). The study clock will start on November 1, 2015. Recruitment will begin February 1, 2016 and will end April 30, 2018. We will enroll an average of 30 participants (who meet study criteria and consent to participate) per month for 27 months, resulting in an enrolled sample size of 800. At this rate of recruitment, we anticipate enrollment of 25% of the sample (200 participants) 7 months into recruitment, which is Month 11 of the proposed project period. Accordingly, we anticipate that 50% enrollment (400 participants) will be achieved by Month 17 of the project period, 75% enrollment (600 participants) will be achieved by Month 23 of the project period, and 100% enrollment will be achieved by Month 30 of the project period.

		Monthly		Cumulative	
**Month**	**Ends**	**Expected**	**Actual**	**Expected**	**Actual**
**4**	2/28/16	20		20	
**5**	3/30/16	30		50	
**6**	4/30/16	30		80	
**7**	5/30/16	30		110	
**8**	6/30/16	30		140	
**9**	7/30/16	30		170	
**10**	8/30/16	30		200	
**11**	9/30/16	30		230	
**12**	10/30/16	30		260	
**13**	11/30/16	30		290	
**14**	12/30/16	30		320	
**15**	1/30/17	30		350	
**16**	2/28/17	30		380	
**17**	3/30/17	30		410	
**18**	4/30/17	30		440	
**19**	5/30/17	30		470	
**20**	6/30/17	30		500	
**21**	7/30/17	30		530	
**22**	8/30/17	30		560	
**23**	9/30/17	30		590	
**24**	10/30/17	30		620	
**25**	11/30/17	30		650	
**26**	12/30/17	30		680	
**27**	1/30/18	30		710	
**28**	2/28/18	30		740	
**29**	3/30/18	30		770	
**30**	4/30/18	30		800	

4C. Screening and Consent Processes

After obtaining consent for the screening phase of the study, trained study research assistants will conduct the screening to determine eligibility for the intervention study and will seek to obtain informed consent. For those who meet eligibility requirements, research staff will carefully explain all aspects of the study to potential participants, including possible benefits and risks,

the schedule of visits, and the expected duration of participation, and will address any questions or concerns regarding the study. Potential participants will be informed that: (1) a decision not to participate in the research will have no impact on their status or expected length of stay at the jail; (2) the study has a Certificate of Confidentiality; (3) confidentiality of study information from jail staff, officers of the court, parole officers or others in the criminal justice system; and (4) and limits of confidentiality, including the jail's mandatory reporting procedures for suicide ideation (see below). The consent form will also state that if an individual does not return to the community from jail, but is sentenced and goes directly to prison instead, s/he is no longer eligible for the study and will not be followed (see Section D2.7 of the grant application). Consent forms will also ask for permission to obtain post-release medical and arrest records even if the participant refuses follow-up interviews. If the participant agrees, s/he will sign an informed consent document and releases of information to obtain medical and arrest records, and will complete the baseline assessment. We will ask participants if they would like us to read the consent forms aloud.

Participants who provide their consent will sign a copy of the document and will be given a signed copy of the informed consent document. Informed consent procedures have been developed to comply with the Code of Federal Regulations 45 CFR 46.116, *General Requirements for Informed Consent* and 46.117, *Documentation of Informed Consent,* and contain all required elements. Freedom to refuse or participate or to discontinue participation at any time without penalty will be emphasized. Once a participant has agreed to participate in the research and signed the informed consent, s/he will undergo the baseline screening assessment.

Attached consent forms include:
- Main study consent form
- Locator forms
- Permission to obtain medical records from the jail
- Permission to obtain medical records from local hospitals

Arrest record releases are not needed because arrest data is self-report data.

4D. Study Procedures (there are no other study protocols)

	**Research procedures** *(all participants)*	**Participant safety procedures** *(all participants; required by ethical obligations to study participants)*	**Clinical intervention** *(SPI only)*
**In jail**	Screening<br>Consent<br>Baseline assessment	Mandatory reporting of severe SI to the jail	Study clinician* will visit participant in jail to conduct the initial SPI session
**~1 month after release**	Post-release follow-up assessment (by phone unless reincarcerated)	Emergency referral based on assessment responses	
**First ~6 months after release**		Emergency referral by SPI clinicians (who are licensed) based on clinical judgment	SPI phone calls (4 planned, another 2 optional) within the first week, and at 1 mo, 3 mo, and 6 mo after jail release

**....4 months after release**	Post-release follow-up assessment (by phone unless reincarcerated)	Emergency referral based on assessment responses	
**....9 months after release**	Post-release follow-up assessment (by phone unless reincarcerated)	Emergency referral based on assessment responses	
**""12 months after release**	Post-release follow-up assessment (by phone unless reincarcerated)	Emergency referral based on assessment responses	

**Study clinicians will be moonlighting for our study. To the extent possible, they will be drawn from the community mental health agencies [redacted] that provide mental health and substance use services to re-entering individuals in each community.*

In addition, we will also be collecting the following non-self-report data:

- Costs to us of providing the treatment
- Facility and community mental health budget information to allow us to project potential costs to them, as well as cost-savings
- Participant hospitalization and other medical records from local area hospitals and emergency rooms
- National death records
- Participant jail medical records (to compare our suicide screening results against the jail's)

4E. End of Participation

At the last (12 months) study assessment, we will:

- Offer to mail all participants information on local suicide, mental health, and substance use resources in their area (this can be mailed with the subject payment).
- Offer to facilitate referrals to treatment, if desired
- Send our assessment results to their current provider in a brief, standardized report, if desired, and if they send us a release of information

Table C2: nmeline	**Year1**				**Year2**				**Year3**				**Year4**			
Hire, train RAs, interventionists																
Recruit, randomize																
Intervene																
Follow-up assessments																
Cata cleaning, analysis, papers																

5. Storage of Data and Samples

5A. Sources of Materials

Research materials collected from participants will include structured clinical interviews and questionnaires. With a written release of information, we will also obtain permission to conduct a records review of participants' medical records at local hospitals. We will also access state and national death registry data for purposes of data collection in the proposed study. Data will be collected at intake and at 1-, 4-, 8-, and 12-month follow-up. All data will be identified only by a study ID number. Names and other identifying information will be kept separate from data collected. Data and material will be collected specifically for the proposed project. During the study, all data collected will be entered into an electronic database and stored in locked and password-protected encrypted files, with only code numbers identifying participants. Any paper files will be kept in a locked filing cabinet. Audio recordings will be stored on a secure sever or kept (on CD) in locked filing cabinets.

5B. End of the Study

At the end of the study, de-identified electronic and paper data will be kept using the same protections as they were kept during the study (i.e., secure research servers, locked filing cabinets in locked research offices). De-identified study data will also be shared as described below.

5C. Plan for Rapid Sharing of Trial Data

After data have been collected and study results published, de-identified data will be made available to other qualified researchers upon request, on a CD or other electronic means compatible with our systems. The request will be evaluated by the PIs to ensure that it meets reasonable standards of scientific integrity. We will also place the de-identified dataset, along with the data dictionary and documentation of data collected, into the NIMH Limited Access Dataset Repository. We have used standardized assessments of suicide ideation, behavior, and attempts (e.g., the C-SSRS), hospitalizations (e.g., medical records) and other outcomes, several of which have been incorporated as common data elements in ongoing NIH common data element initiatives (e.g., Suicide Collection of the PhenX Toolkit), in order to promote data sharing and integration into larger databases and allow other researchers to analyze the data, including conducting meta-analyses.

6. Additional Considerations

NA: No drugs, devices, or gene therapies are used in this study.

7. Risks and Discomforts

7A. Sources of Materials

Research materials collected from participants will include structured clinical interviews and questionnaires. With a written release of information, we will also obtain permission to conduct a records review of participants' medical records at local hospitals. We will also access state and national death registry data for purposes of data collection in the proposed study. Data will be collected at intake and at 1-, 4-, 8-, and 12-month follow-up. All data will be identified only by a study ID number. Names and other identifying information will be kept separate from data collected. Data and material will be collected specifically for the proposed project. All data collected will be entered into an electronic database and stored in locked and password-protected encrypted files, with only code numbers identifying participants. Any paper files will be kept in a locked filing cabinet. Audio recordings will be stored on a secure sever or kept (on CD) in locked filing cabinets.

7B. Potential Risks

There are three major sources of low to moderate risk associated with participation in the proposed study.

1. Potential coercion. It is possible that individuals may feel coerced into participating. This is a particularly important risk to minimize with incarcerated individuals.
2. Increased distress due to assessment or intervention procedures. It is possible that some participants will experience increased intrapersonal or interpersonal psychological distress as a result of participating in assessment or intervention. In the vast majority of cases, we believe that any increased distress experiences will be mild and transitory in nature.
3. Confidentiality and loss of privacy. The greatest potential risks to those participating in the research are legal or social, caused by the inadvertent loss of confidential information obtained during the data collection process. That is, a participant's identity may be inadvertently exposed or questionnaire material may be released or disclosed to unauthorized persons. In the case of such a breach, serious personal and social consequences could conceivably occur. However, such risks can be minimized by instituting the proper procedures to protect confidentiality and by having resources in place to provide counseling and referrals. We have extensive experience taking appropriate measures to safeguard confidential information in research with criminal justice populations. These measures are described below.

7C. Recruitment and Informed Consent

During the second day of incarceration, after medical clearance, jail staff will introduce the study to all inmates and request permission for research staff to approach them. After obtaining consent for the screening phase of the study, research staff will conduct the screening to determine eligibility for the intervention study. After obtaining consent for the screening phase of the study, research staff will conduct the screening to determine eligibility for the intervention study. For those who meet eligibility requirements, the consent process will proceed as described in Section 4C.

7D. Protection Against Risk

All aspects of the study will be conducted in accordance with HIPAA regulations. Data and safety monitoring will take place to assure the safety of subjects (see below). All participants will be reminded that their participation is voluntary and that they can withdraw at any time without penalty. Additionally, the risks described above will be minimized by the following procedures:

1. We will minimize the risk of potential coercion by following standard procedures for obtaining informed consent. We will begin this process during the intake where we will clarify the nature of the study and possible alternatives upfront. Prior to enrolling participants in the research, we will fully explain the study procedures, risks, benefits, and alternatives to participants, emphasizing that participation has no impact on the other services they receive at the jail, the terms or length of their confinement, or any other community services that they receive post-release. All reimbursements for participating will be commensurate with participants' time required for participating in the research.

2. We will minimize the risk of distress. Incarcerated individuals who serve as participants in this research face the risk of increased distress during assessment procedures or study intervention. All participants will be informed that they do not have to answer questions that they find too distressing and will be reminded that they can discontinue participation at any time. Moreover, clinical backup will be provided during all assessments and intervention sessions by a licensed clinician to help facilitate the stabilization and referral process for participants who decompensate during study procedures. This backup will be provided by [redacted] clinicians for assessments or intervention taking place in jail, and by licensed study clinicians for any post-release assessments or interventions taking place in the community. The need for additional services will also be monitored during each clinical (assessment or intervention) contact. Participants will be formally assessed while they are in jail (intake, pre-release) and after release (1-, 4-, 8-, and 12-month assessments). For participant contacts that take place within the jail, participants who report significant homicidal ideation or suicide risk will be referred to appropriate clinical jail staff at baseline for evaluation. Primary clinical coverage for post-release intervention contacts will be provided [redacted]. Primary clinical coverage for all telephone follow-up assessments will be provided through a partnership with the Boys Town National Suicide Hotline, who will set up a dedicated telephone line to provide a "warm transfer" between the study assessor and a mental health crisis counselor, as determined using a detailed implementation protocol (for additional detail see Safety Monitoring Plan), as has been done successfully in [redacted] previous ED-SAFE study. The Boys Town mental health crisis counselor will provide clinical back-up with reporting of dispositions and outcomes back to study staff, using a structured system. Please see Section 8 (Subject Safety Monitoring) for additional details.

3. We will minimize potential risks due to loss of confidentiality of research data by having all information collected and handled by research staff, including study interventionists, trained to deal appropriately with sensitive clinical issues. All participants will be informed about the limits of confidentiality concerning suicidal intent, homicidal intent, suspected child abuse, suspected elder abuse, and jail-required mandatory reporting issues (i.e., sexual contact within the jail, weapons in the jail, jail escape plans). All information will be treated as confidential material and will be available only to research staff. All information will be kept in locked file cabinets at [redacted] and/or [redacted]. Computer data files will be kept on [redacted] and [redacted] secure research servers, will be available only to authorized personnel, and no names or obvious identifying information will be stored in data files. No participant will be identified in any report of the project. Written consent will be obtained to contact other persons for the purpose of locating the participant for follow-up and participants can refuse or revoke such requests. Participants will update their contact information and contact person for the post-release period at each assessment point to ensure that this information remains accurate. To further protect participants, a federal Certificate of Confidentiality will be sought after the grant has been funded. Potential subjects will be informed that a Certificate of Confidentiality has been obtained for this project and that this certificate will protect the investigators from being forced to release any research data in which participants can be identified, even under court order or subpoena, although this protection is not absolute. Potential participants will be informed of the situations in which they may not be protected under

the Certificate of Confidentiality. No information about participants will be released without their permission or where required by law.

Audio recording of intervention sessions is necessary to rate reliability of interview assessments and study clinicians' fidelity to the treatment. As in our past 10 years of research in jails and prisons, audio recording is accomplished through the use of credit-card size digital audio recorders. These digital recordings are regularly transferred to the universities' secure computer servers (designed to hold and protect digital audio and video recordings for clinical trials) via USB connection and secure file transfer to the universities' secure audio/video server, and the recorders are wiped. This is the same procedure that has been used in [redacted] completed and ongoing intervention studies at the [redacted] recruitment site. Participants will be asked to give informed written consent to audio recording at the time of study entry. To assure the confidentiality and protection of participants with respect to audio recording, the following steps will be taken: a) each recording will be labeled with the participant's study identification number, the clinician's/interviewer's name, and the session/interview date; b) all recordings will be stored on a secured computer server designed to hold and protect research data; and c) access to the audio recordings will be limited to research staff who need access to the recordings to perform their duties.

8. Subject Safety Monitoring

As per the research design, there will be research contacts with study subjects during incarceration in the jail and also during the post-release period. Protocols for monitoring and management of subject safety within each context are provided below.

8A. Safety Monitoring Overview

Intervention: Consistent with procedures utilized in our other psychosocial treatment studies for incarcerated individuals and other high risk psychiatric samples, [redacted] will be available by telephone for all study intervention contacts if immediate consultation is needed for an emergent mental health or other problem.

Assessments: Following from [redacted] experience as one of the PIs of the ED-SAFE study, which employed a similar protocol, primary clinical coverage for all follow-up telephone assessments (at months 1, 4, 8, and 12) will be provided by mental health crisis counselors staffed at the Boys Town National Suicide Hotline. Specifically, Boys Town will create a dedicated telephone line through which assessment calls that surpass a specific threshold of risk will be transferred, following a "warm transfer" process between study staff and the mental health crisis counselor at Boys Town. A detailed implementation protocol will be developed such that the thresholds for "warm transfer" to Boys Town will be incorporated into the programming of the REDCap data collection interface, so that decisions to transfer a call will be based upon standardized procedures, thereby reducing the need to solely rely upon human judgment. Similarly, a detailed protocol will be developed so that critical information (i.e., participant location and telephone contact information) will be shared with Boys Town at the time of transfer, and so that Boys Town counselors will have a set of procedures to follow should a call become disconnected. Boys Town will follow a set of procedures for reporting participant dispositions and outcomes back to the study in an official Call Record, within 24 hours of each contact, for purposes of AE and SAE monitoring by the project coordinators and study PIs.

When a member of the research staff is notified of an unanticipated problem, including adverse or serious adverse events, he or she will immediately document the date and details of the event in the Adverse Event Tracking Log. All reporting procedures described below will also be implemented as required. Specific categories of unanticipated problems that require additional action are detailed below. The PIs, [redacted] will be responsible for overseeing the safety of all participants. [redacted] will be available to serve as clinical back-up for [redacted], when required. In all these instances, if the PIs are away, someone will be designated to be in charge of the study for the Universities in their absence. Participant safety will be monitored in two ways: (a) during the intake or ongoing assessments by the research staff, or (b) during intervention sessions. Research assessments will be conducted at baseline, 1-, 4-, 8-, and 12-month follow-up. We will monitor all participants for significant suicidal ideation (SI) and homicidal ideation (HI).

8B. During Incarceration

During incarceration, the standards for mandatory reporting of suicide risk are that we are required to report anyone who has had active suicide ideation while in jail (a "yes" to C-SSRS screener item 2) to the jail. This will include many, though not all, of our study participants (who will report a "yes" to C-SSRS screener item 4, at a minimum, anytime in the past month [though not necessarily while they were in jail]). Participants meeting the jail's SI mandatory reporting criteria (active suicide ideation while in jail, as operationalized by C-SSRS screener item 2) will be referred to a jail mental health clinician for evaluation, who will follow jail procedures. Standard jail procedures include: (1) checking in the jail's electronic medical record

to see if the person has already been flagged as having SI; (2) having a licensed mental health clinician check in with the person and do a suicide risk evaluation; and/or (3) if needed, putting the person on psychiatric observation within the jail. If jail mental health professionals determine someone's risk to go down while s/he is still in jail, that person leaves psychiatric observation and enters the general jail population. If an individual still on psychiatric observation is released on bail, the jail will ask the person picking the detainee up to take him or her to the ED for evaluation. If an individual on suicide psychiatric observation goes to court, the jail provides a letter asking the court to have the person evaluated by a mental health professional before releasing him or her. All of these procedures are set and executed by the jail, which follows its own ethical and legal requirements. We will clearly describe all of this in our consent form. Homicidal risk will be defined by reporting any desire to hurt another to any member of the study staff. Standard jail mandatory reporting procedures (e.g., contact jail mental health staff) will be followed.

<u>8C. In the Community</u>

When participants are in the community, all assessment and intervention contacts will be made by telephone. At the beginning of each call, the rater or clinician will obtain information regarding the participant's location. As licensed clinicians drawn from the existing community mental health care systems, the study clinicians will be responsible for clinical evaluation and emergency referral, should a study participant report any suicidal or homicidal ideation during an SPI intervention contact. The licensed clinician will conduct a suicide and homicide risk assessment to determine whether it is necessary to take immediate action to prevent the participant from causing harm to self or others. If needed, actions that may be taken include having a designated family member or friend transport the person to the closest hospital, or sending local police to escort the person to the nearest hospital to be evaluated for inpatient psychiatric stabilization. [redacted] will be available by telephone for all study intervention contacts if immediate consultation is needed for an emergent mental health or other problem.

As in ED-SAFE (a large, multisite study of suicide prevention after an emergency department visit), if a study participant reports any suicidal or homicidal ideation during a phone assessment contact, study staff will follow standardized procedures to initiate a "warm transfer" to the Boys Town mental health crisis counselor through a dedicated phone line for this study, as described above. These trained counselors will further assess safety risk to determine further actions, to enlist senior staff for more difficult calls, to provide support and information to participants, and to access local service referrals as appropriate. If needed, actions that may be taken include having a designated family member or friend transport the person to the closest hospital, or sending local police to escort the person to the nearest hospital to be evaluated for inpatient psychiatric stabilization. If a call is disconnected during this "warm transfer," the Boys Town mental health crisis counselor will use the contact information provided in an attempt to re-contact the participant, up to 3 separate times. If, after 3 attempts, the mental health crisis counselor is unable to contact the participant, local police will be contacted and dispatched to perform a wellness check. Based upon [redacted] experience in the ED-SAFE study using a similar protocol in collaboration with Boys Town, we anticipate that this latter scenario will occur very infrequently. Within 24 hours of each Boys Town contact, a pdf Call Record will be provided via encrypted email with information regarding the disposition and outcome of each call, for purposes of AE/SAE event monitoring and reporting, and for purposes of data collection (e.g., if a participant was transferred to the Emergency Department) and participant tracking.

It is likely that most suicidal behavior detected during the follow-up assessment will be historical, and therefore will not require a transfer of the call between the blind evaluator and Boys Town.

However, for any *emergent* suicidal ideation or behavior, the blind evaluator will follow the specific protocol for mandatory transfer of calls to Boys Town, as described above. For any *non-emergent, but recent* (i.e., within the past two weeks) suicidal behavior, the blind evaluator will offer to transfer the participant to the Boys Town mental health crisis counselor for additional consultation and referral; this transfer will be voluntary for such participants. In addition to evaluator training on all related protocols, the REDCap database will be programmed to generate prompts, driven by entered assessment data, to further guide the decision making process of blind evaluators with respect to transfer of calls to Boys Town.

8D. Withdrawal from the Study

Subjects will be withdrawn from the study if they request it. If a subject request withdrawal from the study, we will attempt to clarify which part or parts of the study the subject would like to withdraw from (e.g., assessments, intervention, medical record review). If we cannot clarify the subject's intent regarding withdrawal, we will consider the subjects as having withdrawn from the entire study. Whenever possible, their withdrawal will be confirmed in writing. All withdrawal decisions, whether partial withdrawal from procedures or full withdrawal from procedures, will be documented within the REDCap database. There are no other clinical events that would warrant withdrawal. Subjects that are simply lost to follow-up because we were unable to reach them would not be considered "withdrawals." All withdrawals and those lost to follow-up will be included in the analyses, per intention to treat principles.

8E. Criteria for Unblinding

Given that the investigation under study is a psychosocial intervention (i.e., there is no placebo control), and given that community treatment as usual is completely unrestricted in both conditions, there are no *a priori* study criteria for unblinding.

9. Outcome Measures

Assessments will take place at baseline, and at 1, 4, 8, and 12 months post-release. Baseline assessments (including informed consent, locator, and release of information paperwork) will take place in person at the jail; RAs will offer to read each study assessment aloud. Follow-up assessments will take place by telephone (the most feasible way to do follow-ups with this population}, unless a participant is reincarcerated, in which case the follow-up assessment will take place in person at the jail/prison.

Table C1: Assessments	Type	Time (min)	Baseline	1,4,8,12 month FollOW-UD
**Suicidal Ideation and Behavior**				
Columbia Suicide Severitv Ratina Scale (C-SSRS) [181]	Interview	20	X	X
L.I.F.E.[182]-suicidal ideation and behavior	Interview	10		X
Suicide deaths: record review (state/national death reaistrv)	Obiective	0		X
Hospitalizations: Tx History Interview, record review (local hospitals)	Objective	0	X	X
**Psvchiatric Svmotoms:**				
DSM-5 Cross-Cuttina Measure	Self-Reoort	7	X	X
AUDIT and DAST-10	Self-report	7	X	X
**Functionina:** SF-12[183] from RAND Medical Outcomes Study	Self-Report	3	X	X
**Hvoothesized Mechanisms**				
Treatment utilization: Treatment Historv Interview[184]	Interview	8	X	X
Belonainaness: Interoersonal Needs Questionnaire-12[185,186]	Self-Reoort	5	X	4 ,8,12 onlv
Suicide-related oroblem-solvina: Safetv behavior checklist	Self-Reoort	5	X	4 ,8,12 onlv
**Diagnosis:** Mini International Neuropsychiatric Interview (MINI) [187]: **lifetime psychosis, mania/hypomania, major depressive episode, PTSD**	Interview	15		1 mo only
**Total patient time for interview** (minl			**Consent+55**	**65-70**

Primary outcome. Suicide Events is a composite score consisting of the total number of occurrences of any of the following in the year after jail release: (a) attempted suicide (includes suicide deaths}, (b) suicide behaviors (preparatory acts, aborted or interrupted suicide attempts}, as defined using the Columbia criteria,[181,188] and (c) suicide-related hospitalizations. We will use the Treatment History Interview (THI[189]) as well as hospital records to track the number of subsequent hospitalizations and reasons for these admissions. Suicide event data will be assessed using data collected from all possible sources, including follow-up assessments (C-SSRS, THI}, (b) hospital chart reviews, and (c) state/national registries. Data from all sources will be reviewed by 2 research team members for congruence. Disagreements will be reviewed and adjudicated by d . All reports will be classified using C-SSRS criteria. The C-SSRS is the recommende measure of suicidal ideation and behaviors in the NIH PhenX Toolkit as a core data element in all clinical trials for suicide prevention. Although it will not be included in our suicide event composite, we will also track implementation of rescue procedures (e.g. calling Emergency Services/police, breaking confidentiality to inform clinician of high suicide risk) during SPI phone calls or study assessments and compare conditions on this variable.

Secondary outcomes. Suicide Attempts. We will separately assess and evaluate total number of subsequent attempts using the procedures described above. Weeks of active suicidal ideation during the follow-up period will be operationalized using the Longitudinal Interval Follow-Up Evaluation (LIFE;[190]}. At each assessment point, we will ask participants to rate their level of suicidal ideation in the weeks since the last assessment on a 6-point psychiatric status rating scale. This LIFE method yields weekly scores and allows us to examine both the occurrence and *chronicity* of suicidal ideation over the follow-up period. The LIFE calendar will also be used to assess time to first suicide event. As in this study, the LIFE is often administered by phone (i.e.[191,192]). Severity of suicide ideation. We will also assess severity of suicidal ideation using the Suicidal Intensity subscale from the C-SSRS. Psychiatric symptoms will be assessed using the

DSM-5 Level 1 Cross-Cutting Symptom Measure. This measure was chosen because it is the recommended measure of broad psychopathology in the Tier 1 Core for Mental Health research in the NIH PhenX Toolkit, performed well psychometrically in the DSM-5 Field Trials, and is comprehensive yet brief. Substance use will be measured using the Alcohol Use Disorders Identification Test (AUDIT) and the Drug Abuse Screening Test-10 (DAST-10). These measures were chosen as they are well validated, brief measures that have been used in several countries and clinical settings, and are cross-referenced as relevant common data elements in the Suicide Specialty Collection of the NIH PhenX Toolkit. Overall functioning will be measured using the SF-12,[183] a brief, widely used measure of physical and mental health functioning that also provides our secondary cost-effectiveness measure (see below).

Hypothesized mechanisms of SPI effects. We define treatment utilization (*primary*) as the number of outpatient mental health and substance use visits attended in the community in the 3 months prior to baseline or since the last assessment, as indexed by the THI. Belongingness (*exploratory*) will be assessed using the Interpersonal Needs Questionnaire (INQ-12).[185,193] Suicide-related problem-solving (*exploratory*) will be assessed using a standard checklist of suicide safety behaviors and asking whether each was utilized during the most suicidal period since the last interview (identified on the C-SSRS). The checklist includes two subscales to minimize assessment reactivity: the sum of the number of recommended (e.g., call a friend) and the sum of the number of not recommended (e.g., use drugs) responses to suicidal thoughts or urges. These are the most direct, objective measures possible given the time-pressed, chaotic jail setting for baseline assessments and telephone follow-up.

Additional outcomes. Suicide deaths. Given the low incidence of suicide deaths, even in this high risk sample, we do not expect to have sufficient numbers of suicide deaths (separate from the suicide event composite) for meaningful analyses. However, we will track number of suicide deaths using all possible data sources, including hospital records and reviews of state and national death registries. Although our intervention does not target re-arrest directly, it is possible that by increasing service linkage, SPI could reduce re-arrest; therefore, we will assess number of re-arrests and compare conditions on this variable. We will also track days incarcerated in the year after the index release to weight participants by time in the community for other analyses.

Sample descriptors will include baseline demographics. Study inclusion criteria are based on suicide risk rather than diagnosis, but we will gather some basic diagnostic information (lifetime psychosis, mania/hypomania, and major depression) using MINI modules (see Table).[187] We chose the MINI to keep assessment interviews to 1 hour: keeping participant burden minimal (and hence, study procedures feasible and enrollment and follow-up rates high) was our primary consideration. We will administer the MINI modules at 1-mo post-release because: (1) we are assessing lifetime diagnosis, and (2) giving it at the 1-mo follow-up best balances the length of all assessments.

Hospital and death records. Most of [redacted] is covered by 2 large health systems. [redacted] is covered by 3 systems. As in ED-SAFE (a 7-state, 8-site trial [redacted]; see C3)[194], we will obtain releases of information from participants at study intake to conduct chart reviews at all of the hospital systems in each region. As in ED-SAFE, RAs will review charts following a structured protocol, utilizing discharge codes, discharge summaries, medications, laboratory results, operation records, nursing notes, physician progress notes and other notes or comments to determine whether a suicide event occurred. ED-SAFE data showed that this approach is feasible and that it enhanced detection of suicide events over and above telephone follow-up assessment, uniquely identifying 43% of the 1871 detected suicide-related events[195]. Thus, a combination of records review with phone assessment is a feasible and robust approach to detecting suicide-related events. We will also search the National Death Index[196] for ICD-coded suicide deaths in our sample.

Cost-effectiveness measures. Our grant accounting will capture the costs of the SPI

providers. We will also track treatment received as part of SOC for the SPI plus SOC and SOC conditions. SOC (including outpatient, inpatient, and ED mental health and suicide-related medical care visits) will be tracked using the THI, and costs of SOC will be estimated using costs for similar visits to SPI providers and charge data from state hospital and ED data systems adjusted to costs with facility-specific cost-to-charge ratios obtained from Federal cost reports. We will include training costs but exclude other research costs that would not be incurred if SPI were standard care. The primary cost-effectiveness (CE) measure will be the sum of suicide-related hospitalizations and medically treated and fatal suicide acts[197]. Our secondary CE measure will be the SF-12. Our prior work found that Sengupta's HUI3 scoring[198] was the best of 50 scorings of the SF-12. That scoring measures functional status in quality-adjusted life years (QALYs). Costs (and savings) in future years will be discounted to present value in the year of treatment initiation using the 3% discount rate recommended by the Panel on Cost-Effectiveness in Health and Medicine.[199] Costs, benefits will be converted to same-year dollars.

10. Statistical Analysis

<u>10A. Data Analysis</u>

Primary analyses will be *intent-to-treat;* we will examine dose-response effects in secondary analyses. Primary tests will be 2-sided with α=0.05. Site differences will be modeled with fixed effects. Descriptive statistics will include effect sizes and measures of clinical significance (i.e., area under the curve[200]; number needed to treat) for all major comparisons. We will separate primary hypothesis (Aim 1) from remaining hypotheses (Aims 2-4). Standard post hoc procedures will be used to adjust for multiple comparisons when testing secondary hypotheses. <u>There is no planned interim analysis</u>. Analyses will adjust for baseline levels of dependent variables, gender, and yes/no history of suicide attempts. Consistent with CONSORT[201] guidelines, we will pre-specify covariates and will not adjust for imbalance observed post hoc.

Missing Data. We will collect medical record and death record data on all participants. Self-reported count (e.g., suicide events) and historical (e.g., weekly LIFE ratings) data from missed assessments will be gathered at later follow-up assessments. We will use multiple imputation to deal with missing data[202,203]. We will compare treatment conditions on rates of missingness and time to missingness and will test whether baseline characteristics are associated with missingness. Finally, we will perform a sensitivity analysis in which we impute extreme values for missing data to determine the sensitivity of analysis results to missing data.

Outcomes. <u>Primary</u>. We will test the hypothesis that, relative to SOC alone, SPI + SOC will result in fewer suicide events over the 12 month follow-up period, using ordinal logistic regression with lifetime suicide attempts events at baseline as a covariate. The analysis framework will be multivariate ordinal dependent variable regression. We begin with ordinal models because our simulations suggest the count outcome will not likely exceed 3. We will explore using different models such as zero-inflated Poisson or zero-inflated negative binomial with an offset defined by the length of follow-up and time in the community (as opposed to reincarcerated), and other reasonable approaches. Determination of the appropriate modeling will be determined using model selection (information) criteria and the determination will be made blind to the effect of the SIP intervention. <u>Secondary.</u> We will separately test the hypotheses that, relative to SOC alone, SPI + SOC will result in fewer suicide attempts, fewer weeks of active suicide ideation (per the LIFE calendar), lower severity of suicide ideation (C-SSRS scores), longer time to first suicide event, fewer psychiatric symptoms (DSM-5 Cross Cutting Measure scores), and better psychosocial functioning (SF-12 scores). For normally distributed variables (i.e., C-SSRS, DSM-5 Cross Cutting, SF-12, AUDIT, DAST-10), analyses will use a generalized linear mixed model framework for multilevel data (e.g., SAS/proc mixed, HLM) with baseline scores as covariates. For count data (i.e., number of suicide attempts, weeks of active suicide ideation), analyses will use Poisson-class regression methods[204,205] (e.g., negative binomial regression) and will include appropriate tests for zero-inflation and over-dispersion and offset defined by length of time of follow-up and time in the community. Time to suicide event will be analyzed using time-to-event models, beginning with semi-parametric Cox regression models assuming proportionality assumptions are met, otherwise discrete time or parametric continuous time survival models will be used. Model choice will be informed by information criteria and decisions made blind to intervention assignment. <u>Exploratory</u>. Although not part of formal hypotheses, we will also compare conditions on (1) rates of death by suicide, (2) number of re-arrests, and (3) number of emergency referrals generated as part of study assessment safety procedures.

Mechanisms of intervention effects. We will separately test the hypotheses that, relative to SOC alone, SPI + SOC will result in more treatment utilization (number of outpatient mental health and substance use visits as assessed by the THI), more sense of belongingness (INQ-12 Belongingness Scale score), and better suicide-related problem solving (as assessed by the safety behavior checklist), our proposed primary and exploratory mechanisms, using Mplus, which can accommodate both standard and Poisson-class regression methods. We will then test the hypothesis that treatment utilization, suicide-related problem-solving skills, and belongingness (1) predict suicide events, and (2) mediate the effects of SPI on suicide events in

a structural equation model framework to decompose total effects into direct and specific indirect effects. As recommended by MacKinnon et al,[206] the statistical significance of the indirect effect will be assessed using bias-corrected bootstrapped standard errors; 95% CI estimates will be provided.

Predictors/Personalization. We will explore gender, race/ethnicity, lifetime suicide attempts, lifetime highest C-SSRS SI intensity score, severe mental illness (schizophrenia, bipolar disorder), substance use, and # of lifetime arrests as moderators. We expect that SPI is appropriate for a full range of at-risk jail detainees.

Cost-effectiveness analyses. We propose a comparative cost effectiveness (CE) analysis of SPI + SOC relative to SOC. The primary effectiveness measure is the sum of suicide-related hospitalizations and medically treated and fatal suicide acts, with a secondary measure of QALYs (see D2.12). Following widely accepted CE analysis guidelines[199,207], analyses will adopt a societal perspective, considering all economic costs regardless of source. If direct cost savings exceed the program costs, the program is said to offer net cost savings. We describe our statistical plan for determining mean change in and standard deviations of these measures above. The CE ratio equals $\Delta C/\Delta E$, where ΔC is the difference in costs between SPI + SOC and SOC alone and ΔE is the difference in the outcome measure. Using the Crystal Ball add-in to Excel, we will bootstrap 95% confidence intervals around the CE ratio and calculate a cost-effectiveness acceptability curve[208]. Sensitivity analysis will examine CE ratios at 0%, 1% and 5% discount rates.

10B. Expected Attrition and Power Analysis

Attrition. Our target population is pretrial detainees who are returning to the community. We will exclude individuals who expect to be sentenced to prison. However, we expect 6-8% of the pretrial jail detainee participants we consent who do *not* expect to be sentenced to prison will be sentenced anyway. These individuals will not leave jail for the community (i.e., will go directly to prison, not home), meaning that they are not actually eligible for the study, which is a study of suicide prevention in the year after release from pretrial jail detention. Therefore, individuals who go to prison directly from jail rather than back to the community will not be followed, and have been included in our study attrition estimates. This is a standard approach taken in other re-entry studies (e.g., R01 AA021732; U01 DA016191[13]) that must consent participants when their sentencing or release status is still unknown. Sentencing will occur independent of study condition, so their exclusion from analysis (no “at-risk” community months) will be unlikely to influence internal validity. We will follow all remaining participants who are released from jail to the community after the index incarceration through the 12-month post-release period regardless of reincarceration, continued participation in SPI or SOC, or subsequent suicide attempts or hospitalizations. Of the 92-94% of participants who are released from jail to the community after the index incarceration, we conservatively estimate that post-release follow-up rates will be 82% at 1 month, 80% at 4 months, 75% at 8 months, and 70% at 12 months, with 85% of participants providing data for at least one post-release follow-up interview. Post-release follow-up rates in [redacted] previous and ongoing studies have been higher than this. Therefore, we expect that 78% (85% of the 92% who are released from jail) of the 800 enrolled participants will provide evaluable follow-up data. Count (e.g., suicide events) data from missed follow-ups will be gathered at later follow-ups when they occur, and we will collect medical record and death record data on all eligible (i.e., released) participants.

Power. Our primary outcome (suicide events) is a composite of the number of suicide attempts (including suicide deaths), suicide behaviors (per the Columbia criteria), and suicide-related hospitalizations. Previous trials of brief suicide risk reduction interventions in other at-risk populations have yielded relative risks of 1.6, 1.8,[57] 1.8,[58] 2.1,[59] and 2.6[60,61] for suicide attempts (11.0 for suicide deaths[62]), 2.0[60,61] and 3.1[209] for suicide behaviors, and 1.8[60,61] for hospitalization. This study is powered to detect an effect size at the lower end of the range of y/n effect sizes of successful similar studies, relative risk of 1.8 for any attempts, 2.0 for any behaviors, and 1.7 for any hospitalization (see below). In reality, our power will be better because we are measuring total number of each event, not just any event occurrence. ***Base***

rates. The literature provides information about base rates of suicide *deaths* among general populations of jail detainees, but not suicide *events* among jail detainees with suicide ideation. Therefore, we estimated control condition (base rate) event estimates among suicidal jail detainees conservatively as half the rates we observe in ED and inpatient studies.[57,60,61] This conservative estimate given our inclusion criteria of "yes" to C-SSRS item 4 (some suicidal intent) is supported by rates of psychiatric observation (similar to inpatient hospitalization) for suicidality at jail entry (see D2.5). We express ***clinical significance*** using the area under the curve (AUC) statistic, following Kraemer[200]. The AUC is flexible and has a direct and clinically relevant interpretation: the proportion of pairs, sampling one person exposed to the active treatment and another to the control, where the member of the pair exposed to SPI has a more favorable outcome profile. Our expected main effects translate into an AUC of 0.58, indicating that there is a 58% chance that a randomly selected participant from the SOC condition will have more suicide events than a randomly selected person from the SPI condition.[210] This corresponds to a $d=.28$[200], meaning that our study is powered to detect small effects. This is the median effect size for suicide attempts reported in the literature, and we have super-adequate power to detect this effect (96.5%; see below). ***Estimation***. We estimated power using Monte Carlo methods and 1001 replications per condition. Assuming: (1) the outcome is a total count of three outcomes [suicide attempts (including deaths), suicide behavior, and suicide related hospitalization] analyzed with ordinal logistic regression, (2) outcomes are correlated at 0.50 and have base rates of 10%, 18% and 12% in the control group and 5.5%, 9%, and 7% in the SPI group (where these percentages reflect cumulative annual incidence), and (3) a baseline sample of 800 released persons of whom 78% are expected to provide evaluable data; using a type-I error risk of 5%, we will have 96.5% power to detect hypothesized main effects. Power is good but the study is not over-powered given the sensitivity of power to estimated effect sizes: assuming the control condition rates are as estimated, the minimum differences we can detect with 80% power would be SPI condition rates about 6.5% risk of attempts, 10.2% risk of behaviors, and 8% risk of hospitalization. The detectable effect size for ***mediation*** effects range from 0.11 to 0.13 as the correlation of the intervention and the potential mediator ranges from 0.2 to 0.5.[211] Thus, we have power to detect any mediation effect that is clinically significant.

***Non-Completers and Non-Responders*.** Given the unpredictable lives of our target group, flexibility is important in order to make the intervention accessible to them. Participants will *not* be discontinued from the intervention protocol for noncompliance because it has been our experience that recently incarcerated individuals can reengage with providers, even after a period of absenteeism. Participants who report significant suicide or homicidal risk, increased psychiatric symptoms or substance use will be referred to appropriate additional care, but will remain in the research protocol. All participants who are released from jail will be invited to continue all follow-up assessments, and research staff will attempt to maintain regular contact with all participants to collect data at each assessment interval.

11. Human Subjects Involvement and Characteristics

11A. Subject Selection

Participants will be men and women who are 18 years or older who are jailed at the [redacted] or at the [redacted]. Please see Section 3 for a review of the study eligibility criteria and Section 4c for screening and consent procedures.

11B. Jail Detainees as Human Subjects

Because the purpose of this study is to evaluate the effectiveness of SPI for the reduction of suicide risk in the vulnerable months following jail release, it is necessary to sample a jailed population. Jail detainees are an understudied population with complex treatment needs; hence the urgency for more research attending to the mental health and other needs of this population. The project will come under the review of the [redacted] and [redacted] Institutional Review Boards (IRBs), which will ensure compliance with the OHRP Guidance on the Involvement of Prisoners in Research and the requirements of the DHHS regulations 945 CFR, subpart C, and which will apply to OHRP for project certification. We will also obtain a Certificate of Confidentiality.

11C. Sample Composition and Rationale

Extant data from jailed inmates at the [redacted] and [redacted] have been used to estimate the racial and ethnic distribution of the 800 participants in this study. At both jails and nationally, pretrial detainees are 86% male and 14% female; we expect to enroll *at least* 14% women in this study. The overall racial and ethnic distribution for the 500 [redacted] participants will be approximately 24% African American (including individuals who are more than one race, including African American), 18% Hispanic, and 55% non-Hispanic White. The overall racial and ethnic distribution for the 300 [redacted] participants will be approximately 55% African American, 5% Hispanic, and 40% non-Hispanic White. If we recruit 500 participants from [redacted] and 300 from [redacted] as planned, 36% of all participants will be African American, 13% will be Hispanic, and about 50% will be non-Hispanic White. We recognize that NIH separates race and ethnicity and we have done so in the Targeted/Planned Enrollment Table; we are reporting race/ethnicitiy here as the [redacted] and [redacted] report them.

Incarcerated children 18-21 will be eligible. Individuals younger than 18 will be excluded because adolescent detainees are managed through the Juvenile Corrections Divisions and are not placed in adult jail custody. Further, those who are 18 or younger are likely to present with issues and concerns that are different than those who are over the age of 18 (e.g., the type of social support needed, role of parents and guardians, developmental issues). Individuals who are excluded from the study will be treated according to the current [redacted] and [redacted] strategies for jailed individuals, which consists of assessment and psychiatric stabilization as needed.

11D. Efforts to Achieve Targeted Sample Composition

If the minority distribution of our participants falls below the targeted distribution (i.e., if halfway through the study, the proportions of African American or Hispanic participants recruited are less than two-thirds of that minority distribution at the two participating jails), then we will conduct additional outreach to the group that fell below their targeted enrollment numbers. We will also attempt to obtain feedback regarding why individuals for that group may refuse to participate in the study. Based on the feedback, we will take corrective action. We will follow similar procedures for recruitment and enrollment of women. All subjects will be asked to identify their race and ethnicity separately by self-report, at the time of study entry, when demographic information is collected. We plan to conduct analysis to determine whether

minority status is associated with any of the primary or secondary outcome data collected as part of this investigation.

11E. Safeguards for Vulnerable Populations

Safeguards for vulnerable populations have been delimited in Sections 7, 8, and 15. As they are not relevant to the current study, there will be no safeguards specific to durable power of attorney, pregnancy testing, contraception use, and no Human Subjects Protection Unit involvement.

11F. Qualifications of Investigators

Investi ator	Role	Qualifications
Jennifer Johnson, PhD	MPI	[redacted]
Lauren Weinstock, PhD	MPI	[redacted]
[redacted]		[redacted]
[redacted]	[redacted]	[redacted]
[redacted]		[redacted]
[redacted]		[redacted]
[redacted]	[redacted]	[redacted]
[redacted]		[redacted]
[redacted]	[redacted]	[redacted]
[redacted]		[redacted]
[redacted]	[redacted]	[redacted]
[redacted]	[redacted]	[redacted]

[redacted]	[redacted]	[redacted]
[redacted]	[redacted]	[redacted]

12. Anticipated Benefit

12A. Benefits of the Proposed Research to the Subjects and Others

The potential risks associated with participation in this study appear to be mild to moderate. Although there is a risk for distress, the procedures proposed for monitoring distress should ensure that participants who require a higher level of care receive it. Participants assigned to SPI may benefit from reduction in suicide risk and improvement in post-release engagement with community treatment and overall functioning. The study provides additional screening, assessment, and referral to emergency services, as needed, for all study participants, and in no way restricts or limits the treatment subjects would have received had they not participated in the study. Moreover, this study is likely to yield generalizable knowledge that will be used to improve suicide prevention intervention and services for other incarcerated individuals. Thus, the potential benefits outweigh the potential risks of this study.

13. Classification of Risk *(for the study as a whole)*

There are two sets of human subjects issues to consider when undertaking a study with the goal of evaluating an intervention aimed at reducing suicidal behavior around the time of jail detention. The first set of issues relates to the overall risk of the population under study, regardless of the decision to consent to participate in a study. The second set of issues relates to possible additional risk assumed by participation in research.

13A. Naturalistic Risk of the Population Under Study

As a function of the study aims and inclusion criteria, the population under study is expected to be at risk for suicide attempt or reattempt, both fatal and nonfatal. Second, individuals at risk for suicide often have several comorbid conditions, including severe mental illness and substance use behaviors, which can place them at risk for psychiatric hospitalization and rehospitalization, substance use overdose, inpatient detoxification, and residential substance use treatment. It is also anticipated that a substantial proportion of study participants will have been exposed to interpersonal violence in the community, placing them at future risk for IPV. Finally, as we will be recruiting from the jail setting, there is also risk of rearrest and reincarceration. In general jail populations (not even those judged to be at risk for suicide), prevalence rates are:

- Mental health problems: 56%
- Substance use problems: 66%
- Violent victimization: ~50%
- Rearrest within 12 months: ~30%

We anticipate that naturally occurring rates of these conditions will only be higher in a jail sample selected to be at high suicide risk. These risks exist regardless of whether individuals choose to consent to be research participants, and exist regardless of randomization to SPI or SOC.

13B. Potential Risks Associated with Study Participation

As reviewed in Section 7, there are three major sources of low to moderate risk associated with participation in the proposed study: 1) Potential coercion, 2) distressing assessment, and 3) loss of privacy. There are several protocols in place to minimize these potential risks, also reviewed in Section 7. Individuals without consent capacity will not be enrolled in this study.

13C. Overall Risk and Benefit Consideration

The potential benefits of identifying effective treatments for suicidal patients appear to outweigh the potential risks of this study. Improvements in assessment and treatment of jail detainees at risk for suicide are urgently needed. Further, evaluation of suicide prevention interventions in any high-risk population may yield important benefits for multiple at-risk groups. This, study results will have important implications for a variety of stakeholders, including affected individuals, family members, healthcare providers, managed care organizations, health insurers, administrators, and policy makers.

The major risk, that of adverse events, should not be increased by study participation and in fact should be reduced by the enhanced monitoring and risk reduction procedures delineated in this protocol.

14. Consent Documents and Process

14A. Designation of Those Obtaining Consent

Trained research assistants designated as able to obtain consent (see Section 4) will obtain informed consent. As this study does not enroll participants younger than age 18 years, there is no procedure to obtain assent from minors.

14B. Consent Procedures

Consent procedures are outlined in Section 4C.

14C. Consent Documents

The consent form contains all required elements (consent document attached). There is only one consent document for participant enrollment into this study.

15. Data and Safety Monitoring (Includes Quality Assurance Procedures)

15A. Data and Safety Monitor

The Principal Investigators, together with the Co-Investigators, Site Monitor, and Safety Officer, will be responsible for monitoring the safety of this trial, executing the Data and Safety Monitoring Plan, and complying with external reporting requirements. External data and safety monitoring will be conducted by:

[redacted] Institutional Review Board: Through an Institutional Authorization Agreement with [redacted] (effective 08/20/2015), the [redacted] Institutional Review Board (IRB) will be the IRB of record for this study. Subject recruitment will not begin until approval is obtained from the [redacted] IRB. IRB approval is required for the study protocols plus any amendments, informed consent forms, and subject information sheets.

NIMH Data Safety and Monitoring Board: A DSMB created by NIMH will monitor and evaluate the safety of the participants through the course of the research study. The DSMB will receive a report 3 times per year, unless requested otherwise. NIMH will provide a DSMB report template. This report will include a Study Overview, Consort Chart, Enrollment Table, a Treatment Discontinuation Table, Demographics, a Table of Suicide Attempts – by subject site, method, a brief narrative for each suicide attempt – and a Summary Table of all adverse events (AEs), serious adverse events (SAEs) (including those non-suicide related SAEs), and unanticipated problems (UPs). Tables reporting on protocol and data integrity will also be provided (e.g., protocol deviations and violations, and tracking rates of missing forms and resolved data queries). Dedicated personnel will be hired for the purpose of preparing DSMB reports (DSMB Reporter) and for review of DSMB reports (Safety Officer). These personnel will be based out of the [redacted] site so as to not directly interface with [redacted]-based follow-up evaluators, in an effort to preserve the evaluator blind.

15B. Data Monitoring Plan

Data Safety and Storage: Data management and data entry will be conducted by an experienced team, led by [redacted] (Co-I), that will establish a participant tracking and data monitoring system within REDCap (Research Electronic Data Capture; hosted through [redacted] secure servers). REDCap is a secure web application designed to support data capture for research studies, providing user-friendly web-based case report forms, real-time data entry validation (e.g., for types of data and range checks), audit trails, and a de-identified data export mechanism to common statistical packages (SPSS, SAS, Stata, R/S-Plus). The system was developed by a multi-institutional consortium and was initiated at Vanderbilt University. A local REDCap server is hosted by the [redacted]. Network transmissions (data entry, web browsing, etc) in REDCap are protected via Secure Sockets Layer (SSL) encryption. REDCap data collection projects rely on a thorough study-specific data dictionary defined in an iterative self-documenting process by appropriate members of the research team, with planning assistance from the [redacted]. REDcap provides a secure, web-based application that is flexible enough to be used for a variety of types of research, provide an intuitive interface for users to enter data and have real time validation rules at the time of entry. Access to data stored on REDCap can be restricted at different levels, as needed (e.g., research assistant, PI, Co-Is). Exported data from REDCap will be stored on the [redacted] secure password-protected server. The study-specific REDCap database will be backed-up automatically on a daily basis to the FileServer hard disk. Backup files will be both compressed

and encrypted during this transmission. The File Server network drives will be backed-up to tapemedia daily (incremental) and weekly (full backups). Locally stored tapes will be moved off site on a weekly basis. All backup tapes will be stored in a locked, secure environment.

Data from any paper records (i.e., those collected within the jails) will be entered into REDCap, and paper records will be stored in locked file cabinets within locked offices at [redacted] and [redacted]. To ensure reliability and validity of interview assessments and intervention sessions, all interviews, assessments, and sessions will be – with participant consent – audio recorded. Audio files will identify each participant by ID number only, and will be stored on the [redacted] secure password-protected server. Neither data nor reports will contain any identifying information.

Data Integrity: Data quality will be reviewed continuously, and monitored by random inspection of completed forms and databases by one of the research assistants, with any problems detected discussed with the PIs. Standard data checking procedures will include checking forms for missing data, double entry with discrepancy resolution, daily backups of computer files, and examination of key variables for skewness, variability, missing data, and outliers. Monthly reports on the status of recruitment, completeness of records, and completeness of data fields will be generated and reviewed at the monthly Scientific Advisory Team meetings with all study investigators. Summaries will also be shared with the Site Monitor prior to each Site Review Meeting. Adjustments to the data collection schedule and monitoring of staff may be indicated following these reviews. In addition, major study variables will be operationalized and distributions will be displayed in figures and tables to ensure completeness and usability of collected information, and identify corrections for out-of-range values, missing data, or other procedural issues.

Data Access: Only study staff will have access to data. No confidential information may be released outside the study team without the express written consent of the study participants, unless mandatory for child and elder abuse and in situations in which the risk of suicide or homicide is imminent.

Educational Training: All study staff will have undergone mandatory education in human subjects' research protections. As detailed previously, all study staff will undergo specific training relevant to their study roles and responsibilities.

15C. Site Monitoring Plan

In accordance with NIH policies, one independent monitor will be assigned to conduct routine site monitoring for this study. The Site Monitor, who will not be a member of the study team, will visit both the [redacted] and [redacted] research offices every six months, and will meet with the study PIs following these biannual site visits either in person or via routine conference call to review adherence to the principles of good clinical practices (GCP). Regulatory documents will be maintained in the study Regulatory Binder, which will be reviewed by the Site Monitor prior to each Site Review Meeting (SRM). In addition, the Site Monitor will review compliance with the IRB protocol and informed consent requirements and data integrity prior to each SRM.

Monitoring for compliance with the IRB protocol and informed consent documents

Every six months, the respective project coordinators at [redacted] and [redacted] will randomly select a subset of cases (10% of newly enrolled participants since the last audit) for an internal study audit, in order to assess degree of compliance with essential IRB requirements, including randomization procedures (e.g., allocation concealment), and with established informed consent procedures. This audit will also include a review of all adverse event reports since the prior audit. Should significant problems with compliance be detected through the audit process, then

corrective actions will be immediately taken, including more intensive training and supervision of relevant study staff members and/or establishing a new process of routine monitoring of specific areas of compliance. Information gathered from ongoing internal audits will be summarized in a written report, which the Site Monitor will review prior to the SRM. During this meeting, the Site Monitor will also review any problems with compliance that were detected at the last audit, as well as corrective action taken and results of that action. The Site Monitor will determine whether actions have been sufficient or if any further change is necessary. Any significant issues regarding problems with compliance will be included in the Site Monitor's meeting report, which will be shared with the IRB and NIMH CTOBB.

15D. Safety Monitoring Plan

The protocol for monitoring subject safety is delineated in Section 8 (Subject Safety Monitoring). The Principal Investigators and the Co-Investigators will be responsible for monitoring and reporting adverse events, reviewed by the study Safety Officer and submitted to the [redacted] IRB and NIMH DSMB within the appropriate timeframes. As specified in Section 13, there are several naturalistic outcomes (e.g., violent victimization, rearrest) that can be expected as a function of the population under study, yet are not direct targets of the intervention under study. As this trial is focused on suicide prevention, the definitions provided below are specific to suicide prevention outcomes and proximal related risks (e.g., psychiatric and substance use hospitalizations).

<u>Adverse Events Definitions:</u>

<u>Adverse Event</u> – any unfavorable and unintended sign, symptom or disease temporally associated with the use of a medical or behavioral treatment or intervention regardless of whether it is considered related to the treatment or intervention.

<u>Expected Adverse Event</u> – an event that may be reasonably anticipated to occur as a result of the study procedure and is described in the consent form.

<u>Unexpected Adverse Event</u> – any adverse event which is not described in the consent form and is unanticipated. An event that might have been anticipated but is more serious than expected or occurs more frequently than expected, would be considered an unexpected adverse event.

<u>Serious Adverse Event</u> – (21 CFR 312) includes any untoward medical occurrence that at any dose results in death or the immediate risk of death, hospitalization or prolonging of an existing hospitalization, persistent or significant disability/incapacity or a congenital anomaly/birth defect (NIH guide-6/11/99).

<u>Study Definitions</u>

Specifically, we will consider the following events Serious Adverse Events (SAE):

a. Death for any reason;
b. A suicide attempt, defined as any action taken with intent to die, as stated by the patient or noted in the medical record;
c. Inpatient hospitalization, suicide- or potentially suicide-related (e.g., all mental health or substance use-related hospitalizations);

Additionally, we will consider the following events non-serious Adverse Event (AE):

a. Evidence of coercion to participate;
b. Distress during the assessments;
c. Access of confidential information by a non-authorized person; and
d. Non-suicidal self-harm

<u>Severity</u>

Each adverse event will be graded in terms of severity:

a. Non-severe adverse event
b. Severe adverse event resulting in hospitalization or prolongation of existing hospitalization, a persistent or significant disability/incapacity.
c. Life-threatening adverse event
d. Fatal adverse event

<u>Change in Status</u>

For SAEs, change in status will be documented:

<u>No change in status</u>: if SAE occurs in an individual with a lifetime history of respective event at time of study enrollment (e.g., suicide attempt in an individual with lifetime history of suicide attempt prior to participation)

<u>Change in status</u>: SAE represents first onset in an individual without lifetime history of respective event at time of study enrollment (e.g., first onset suicide attempt in an individual without lifetime history of suicide attempt prior to participation)

<u>Attribution</u>

For each adverse event, one of the following attributions is assigned:

Definite:	Adverse event is clearly related to intervention
Probable:	Adverse event is likely related to intervention
Possible:	Adverse event may be related to intervention
Unlikely:	Adverse event is doubtfully related to intervention
Unrelated:	Adverse event is clearly not related to intervention

<u>Reporting</u>

Considering the nature of the study, we expect serious adverse events to occur, including suicide attempts. However, given the nature of the study, it is unlikely that they will be related to the study procedures. The other adverse events that are possible, including inadvertent disclosure of protected health information, have been described in Risks and Discomforts. If any of these adverse events occur, or any other unanticipated events that are identified, the following procedure will be activated:

The research staff member who observes or is notified of an adverse event (e.g., distress during the baseline assessment) will notify the Principal Investigators on the same business day. The PIs or their designee will complete an Adverse Event Form for each event, and will determine if the event is an SAE. SAEs will be forwarded within 24 hours to the Safety Officer for review and final sign-off. This signed and reviewed form will then be sent by email to the [redacted] IRB and NIMH CTOBB for review within 72 hours of the event being reported to the study team. Any SAE (unanticipated or anticipated), whether or not considered related to the intervention, will be reported within this timeframe. Summary reports of adverse events will be provided to the [redacted] IRB and NIMH DSMB 3 times per year, unless requested otherwise.

The Principal Investigators will have regular meetings with staff and personnel to discuss the impact of participation upon patients. If concerns are reported, the PI and Co-Investigators will discuss the issues with the IRB and DSMB and will decide if changes are warranted.

<u>15E. Interim Analysis Plan</u>

Because the study is not powered for interim analyses, no interim analyses are planned. This study was powered accordingly.

16. Alternative Therapies

The consent form states: “During your participation in this research, you are free to receive other treatments or services. There are many other forms of treatment available in jail and especially in the community after release.”

17. Confidentiality

We will minimize potential risks due to loss of confidentiality of research data by having all information collected and handled by research staff, including study interventionists, trained to deal appropriately with sensitive clinical issues. All participants will be informed about the limits of confidentiality concerning suicidal intent, homicidal intent, suspected child abuse, suspected elder abuse, and jail-required mandatory reporting issues (i.e., sexual contact within the jail, weapons in the jail, jail escape plans). All information will be treated as confidential material and will be available only to research staff. All information will be kept in locked file cabinets at [redacted] and/or [redacted]. Computer data files will be kept on [redacted] and [redacted] secure research servers, will be available only to authorized personnel, and no names or obvious identifying information will be stored in data files. No participant will be identified in any report of the project. Written consent will be obtained to contact other persons for the purpose of locating the participant for follow-up and participants can refuse or revoke such requests. Participants will update their contact information and contact person for the post-release period at each assessment point to ensure that this information remains accurate.

To further protect participants, a federal Certificate of Confidentiality will be sought after the grant has been funded. Potential subjects will be informed that a Certificate of Confidentiality has been obtained for this project and that this certificate will protect the investigators from being forced to release any research data in which participants can be identified, even under court order or subpoena, although this protection is not absolute. Potential participants will be informed of the situations in which they may not be protected under the Certificate of Confidentiality. No information about participants will be released without their permission or where required by law.

Audio recording of intervention sessions is necessary to rate reliability of interview assessments and study clinicians' fidelity to the treatment. As in our past 10 years of research in jails and prisons, audio recording is accomplished through the use of credit-card size digital audio recorders. These digital recordings are regularly transferred to the universities' secure computer servers (designed to hold and protect digital audio and video recordings for clinical trials) via USB connection and secure file transfer to the universities' secure audio/video server, and the recorders are wiped. This is the same procedure that has been used in [redacted] completed and ongoing intervention studies at the [redacted] recruitment site. Participants will be asked to give informed written consent to audio recording at the time of study entry. To assure the confidentiality and protection of participants with respect to audio recording, the following steps will be taken: a) each recording will be labeled with the participant's study identification number, the clinician's/interviewer's name, and the session/interview date; b) all recordings will be stored on a secured computer server designed to hold and protect research data; and c) access to the audio recordings will be limited to research staff who need access to the recordings to perform their duties.

After data have been collected and study results published, de-identified data will be made available to other qualified researchers upon request, on a CD or other electronic means compatible with our systems. The request will be evaluated by the PIs to ensure that it meets reasonable standards of scientific integrity. We will also place the de-identified dataset, along with the data dictionary and documentation of data collected, into the NIMH Limited Access Dataset Repository, and if relevant, other appropriate federal research data repositories.

18. Conflict of Interest

NIH guidelines on conflict of interest have been distributed to all investigators.

There are no conflicts of interest to report. Non-NIH investigators will abide by the conflict of interest policies of their own institutions.

19. Research and Travel Compensation

Travel compensation is not provided because participants do not travel. Interviews during incarceration occur at the jail/prison facility. Interviews after release take place by phone.

Research compensation. To ensure compliance with assessments, each participant will be paid a fee for his or her time at each follow-up (post-jail release) assessments. Because we do not want participants to volunteer for the intake assessment for financial reasons, we will not reimburse participants for the intake assessment. Follow-up (post-jail release) assessments will take place by phone. For participation on each follow-up assessment, participants will receive $60 money order, which will be mailed to them after each follow-up assessment. If a participant is reincarcerated, that person can: (1) let us know when they get out and have us mail it to them then, or (2) have us mail it to a friend or family member.

20. References

1. Minton T. *Jail inmates at midyear 2012 - statistical tables.* Bureau of Justice Statistics;2013.
2. Pew Center on the States. *One in 100: Behind bars in America 2008.* Washington, D.C.: The Pew Charitable Trust; 2008.
3. Walmsley R. *World prison population list.* London: King's College London International Centre for Prison Studies;2009.
4. James D, Glaze, LE. *Mental Health Problems of Prison and Jail Inmates.* Bureau of Justice Statistics Special Report;2006.
5. Jordan BK, Schlenger, W.E., Fairbank, J.A., Caddell, J.M. Prevalence of psychiatric disorder among incarcerated women: Convicted felons entering prison. *Archives of General Psychiatry.* 1996;53:513-519.
6. Teplin LA, Abram, K.M., McClelland, G.M. Prevalence of psychiatric disorders among incarcerated women, I: Pretrial jail detainees. *Archives of General Psychiatry.* 1996;53:505-512.
7. Fazel S, Baillargeon, J. The health of prisoners. *The Lancet.* 2011;377(9769):956-965.
8. Fazel S, Bains, P, Doll, H. Substance abuse and dependence in prisoners: a systematic review. *Addiction.* 2006;101:181-191.
9. Lurigio AJ. Effective services for parolees with mental illnesses. *Crime & Delinquency.* 2001;47(3):446-461.
10. Crilly JF, Caine, E.D., Lamberti, J.S., Brown, T., Friedman, B. Mental health services use and symptom prevalence in a cohort of adults on probation. *Psychiatric Services.* 2009;60(4):542-544.
11. Lurigio AJ, Cho, Y.I., Swartz, J.A., Johnson, T.P., Graf, I., Pickup, L. Standardized assessment of substance-related, other psychiatric, and comorbid disorders among probationers. *International Journal of Offender Therapy and Comparative Criminology.* 2003;47(6):630-652.
12. Friedmann P, Taxman, FS, Henderson, CE. Evidence-based treatment practices for drug-involved adults in the criminal justice system. *Journal of Substance Abuse Treatment.* 2007;32:267-277.
13. Friedmann P, Katz E, Rhodes A, et al. Collaborative behavioral management for drug-involved parolees: Rationale and design of the Step'n Out study. *Journal of Offender Rehabilitation.* 2008;47(3):290-318.
14. Clarke J, Hebert, MR, Rosengard, C., Rose, JS, DaSilva, KM, Stein, MD. Reproductive Health Care and Family Planning Needs Among Incarcerated Women. *American Journal of Public Health.* 2006;96:834-839.
15. Hale G, Oswalt, KL, Cropsey, KL, Villalobos, GC, Ivey, SE, Matthews, CA. The contraceptive needs of incarcerated women. *Jurnal of Womens Health (Larchmont).* 2009;18(8):1221-1226.
16. Guerino P HP, Sabol WJ. *Prisoners in 2010.* U.S. Bureau of Justice Statistics;2011.
17. The_Current_Moment. Mass Incarceration Follow up. *On the politics and economics of the present* 2012; http://thecurrentmoment.wordpress.com/2012/06/01/mass-incarceration-follow-up/. Accessed 6-13-13.
18. Hayes LM, & Rowan, J. R. . *National study of jail suicides: Seven years later.* . Mansfield, MA: National Center for Institutions and Alternatives; 1988.

19. Charles DR, Abram, K. M., McClelland, G. M., & Teplin, L. A. Suicidal ideation and behavior among women in jail. *Journal of Contemporary Criminal Justice.* 2003;19:65-81.
20. Sarchiapone M, Jovanovic, N, Roy, A, Podlesek, A, Carli, V, Amore, M, Mancini, M, Marusic, A. Relations of psychological characteristics to suicide behavior: Results from a large sample of male prisoners. *Personality and Individual Differences.* 2009;47(4):250-255.
21. DuRand CJ, Burtka, G. J., Federman, E. J., Haycox, J. A., & Smith, J. W. A quarter century of suicide in a major urban jail: Implications for community psychiatry. *American Journal of Psychiatry.* 1995;152(7):1077-1080.
22. Fazel S, Benning, R. Suicides in female prisoners in England and Wales, 1978-2004. *The British Journal of Psychiatry.* 2009;194:183-184.
23. Daniel A, Fleming J. Serious suicide attempts in a state correctional system and strategies to prevent suicide. *The Journal of Psychiatry and Law.* 2005;33:227-247.
24. Minton TD. Jail inmates at midyear 2010 - Statistical tables. In: Bureau of Justice Statistics, ed. Washington, DC: U.S. Department of Justice; 2011.
25. US_Department_of_Justice_Criminal_Justice_Information_Services_Division. Crime in the United States 2012. 2014; www.fbi.gov/about-us/cjis/ucr/crime-in-the-u.s/2012/crime-in-the-u.s.-2012/persons-arrested/persons-arrested. Accessed 6-5-14, 2014.
26. Sabol W, Minton TD. Jail inmates at midyear 2007. Report No. NCJ 221945. Washington, D.C.: Bureau of Justice Statistics; 2008.
27. Guerino P, Harrison PM, Sabol WJ. Prisoners in 2010. Report No. NCJ 236096. . Washington, D.C.: U.S. Bureau of Justice Statistics; 2011.
28. Solomon AL, Osborne JWL, LoBuglio SF, Mellow J, Mukamal DA. Life after lockup: Improving reentry from jail to the community. Washington, DC: Urban Institute, Justice Policy Center; 2008.
29. Center for Substance Abuse Treatment. Substance Abuse Treatment for Adults in the Criminal Justice System. Treatment Improvement Protocol (TIP) Series 44. DHHS Publication No. (SMA) 05-4056. . Rockville, MD: Substance Abuse and Mental Health Services Administration: 2005.
30. Woff N, Plemmons, D, Veysey, B, Brandli, A. Release planning for inmates with mental illness compared with those who have other chronic illnesses. *Psychiatr Serv.* 2002;53(11):1469-1471.
31. Leukefeld A, Brower J. Basics and Beyond: Suicide Prevention in Jails. In: Corrections NIo, ed: United States Department of Justice; 2012.
32. Mumola CJ. Suicide and homicide in state prisons and local jails. In: Statistics BoJ, ed: United States Department of Justice; 2005.
33. Wilkins N, Thigpen S, Lockman J, et al. Putting program evaluation to work: a framework for creating actionable knowledge for suicide prevention practice. *Translational behavioral medicine.* 2013;3(2):149-161.
34. US_Marshalls. Suicide Prevention in Jails: Basics and Beyond. http://www.usmarshals.gov/prisoner/jail_suicide.pdf. Accessed 1-21-14.
35. Daniel A. Preventing suicide in prison: A collaborative responsibility of administrative, custodial, and clinical staff. *J Am Acad Psychiatry Law.* 2006;34(2):165-175.

36. Hayes LM. National Study of Jail Suicide: 20 years later. In: National Institute of Corrections JD, ed: United States Department of Justice; 2010.
37. Kariminia A, Law MG, Butler TG, et al. Suicide risk among recently released prisoners in New South Wales, Australia. *The Medical journal of Australia.* 2007;187(7):387-390.
38. Pratt D, Piper M, Appleby L, Webb R, Shaw J. Suicide in recently released prisoners: a population-based cohort study. *Lancet.* 2006;368(9530):119-123.
39. Stewart LM, Henderson CJ, Hobbs MS, Ridout SC, Knuiman MW. Risk of death in prisoners after release from jail. *Australian and New Zealand journal of public health.* 2004;28(1):32-36.
40. Zlodre J, Fazel S. All-cause and external mortality in released prisoners: systematic review and meta-analysis. *American journal of public health.* 2012;102(12):e67-75.
41. Keaveny M, Zauszniewski, JA. Life events and psychological well-being in women sentenced to prison. *Issues in Mental Health Nursing.* 1999;20(1):73-89.
42. Hurley W, & Dunne, M. P. Psychological distress and psychiatric morbidity in women prisoners. *Australian and New Zealand Journal of Psychiatry.* 1991;25:461-470.
43. Richie BE. Challenges incarcerated women face as they return to their communities: Findings from life history interviews. *Crime Delinquency.* 2001;47:368-389.
44. Kellett NC, Willging, C.E. Pedagogy of individual choice and female inmate reentry in the U.S. Southwest. *International Journal of Law and Psychiatry.* 2011;34(11):256-263.
45. Nelson M, Deess, P, Allen, C. The first month out: Post-incarceration experiences in New York City. *Federal Sentencing Reporter,.* 2011;24(1):72-75.
46. Chandler RK, Fletcher, B. W., & Volkow, N. D. Treating drug abuse and addiction in the criminal justice system: Improving public health and safety. *JAMA.* 2009;301(2):183-190.
47. Harris RM, Sharps, P.W., Allen, K., Anderson, E.H., Soeken, K., Rohatas, A. The interrelationship between violence, HIV/AIDS, and drug use in incarcerated women. *Journal of the Association of nurses in AIDS care.* 2003;14(1):27-40.
48. Johnson JE, Schonbrun, Y. C., Nargiso, J. E., Kuo, C. C., Shefner, R. T., Williams, C. A., & Zlotnick, C. “I know if I drink I won’t feel anything”: Substance use relapse among depressed women leaving prison. *International Journal of Prisoner Health.* . 2013;9(4):1-18.
49. Johnson J, Schonbrun, YC, Peabody, ME, Shefner, RT, Fernandes, KM, Rosen, RK, Zlotnick, C. Provider experiences with prison care and aftercare for women with co-occurring mental health and substance use disorder: Treatment, resource, and systems integration challenges. under review.
50. Mumola C, Karberg JC. Drug use and dependence, state and federal prisoners, 2004. Report No. NCJ 213530. Washington, D.C.: Bureau of Justice Statistics; 2006.
51. Binswanger IA, Stern MF, Deyo RA, et al. Release from prison--a high risk of death for former inmates. *The New England journal of medicine.* 2007;356(2):157-165.
52. Bird SM, Hutchinson SJ. Male drugs-related deaths in the fortnight after release from prison: Scotland, 1996-99. *Addiction.* 2003;98(2):185-190.
53. Krinsky CS, Lathrop SL, Brown P, Nolte KB. Drugs, detention, and death: a study of the mortality of recently released prisoners. *The American journal of forensic medicine and pathology.* 2009;30(1):6-9.
54. Zlodre J, Fazel, S. All-cause and external mortality in released prisoners: Systematic review and meta-analysis. *American Journal of Public Health.* 2012;102:e67-e75.

55. Haglund A, Tidemalm, D, Jokinen, J., Langstrom, N, Lichetenstein, P, Fazel, S, Runeson, B. Suicide after release from prison: A population-based cohort study From Sweden. *Journal of Clinical Psychiatry.* 2014;75(10):1047-1053.
56. While D, Bickley, H, Roscoe, A, Windfuhr, K, Rahman, S, Shaw, J, Appleby, L, Kapur, N. Implementation of mental health service recommendations in England and Wales and suicide rates, 1997–2006: a cross-sectional and before-and-after observational study. *Lancet.* 2012;DOI:10.1016/S0140-6736(11)61712-1.
57. Vaiva G, Ducrocq, F, Meyer, P, Mathieu, D, Philippe, A, Libsersa, C, Goudemand, M. Effect of telephone contact on further suicide attempts in patients discharged from an emergency department: Randomised controlled study. *BMJ.* 2006;332:1241.
58. Carter G, Clover, K, Whyte, IM, Dawson, HA, Este, CD. Postcards from the EDge project: randomised controlled trial of an intervention using postcards to reduce repetition of hospital treated deliberate self-poisoning. *BMJ.* 2005;331:805.
59. Vijayakumar L, Umamaheswari, C, Ali, ZSS, Devaraj, P, Kesavan, K. Intervention for suicide attempters: A randomized controlled study. *Indian J Psychiatry.* 2011;53(3):244-248.
60. Weinstock L, Gaudiano, B, Melvin, C, Miller, I. Extending the Coping Long Term with Active Suicide Program to Bipolar Disorder: A Pilot Randomized Study. . Association for Behavioral and Cognitive Therapy; November, 2013; Nashville.
61. Miller I, Gaudiano, B, Weinstock, L, Armey, M. The "Coping Long Term with Attempted Suicide Program (CLASP): Pilot Randomized Trial with Patients with Major Depression. Association for Behavioral and Cognitive Therapy; November, 2013; Nashville, TN.
62. Fleischmann A, Bertolote, JM, Wasserman, D, De Leo, D, Bolhari, J, Botega, NJ, De Silvan, D, Phillips, M, Vijayakumar, L, Varnik, A, Schlebusch, L, Thanh, HTT. Effectiveness of brief intervention and contact for suicide attempters: A randomized controlled trial in five countries. *Bulletin of the World Health Organization.* 2008;86(9):703-709.
63. O'Brien P. *Making it a Free World: Women in Transition from Prison.* New York: State University of New York Press; 2001.
64. SAMHSA SAaMHSA. Substance abuse treatment for women offenders: Guide to promising practices. In: Services USDoHaH, ed: Center for Substance Abuse Treatment, Rockville, MD; 1999.
65. Hills HA. *Creating effective treatment programs for persons with co-occurring disorders in the justice system (GAINS Center monograph).* Delmar, NY: The GAINS Center; 2000.
66. Browne A, Miller B, Maguin E. Prevalence and severity of lifetime physical and sexual victimization among incarcerated women. *International Journal of Law and Psychiatry.* 1999;22(3-4):301-322.
67. Freudenberg N. Adverse effects of US jail and prison policies on the health and well-being of women of color. *American Journal of Public Health.* 2002;92(12):1895-1899.
68. Fickenscher A, Lapidus J, Silk-Walker P, Becker T. Women behind bars: Health needs of inmates in a county jail. *Public Health Reports.* 2001;116:191-196.
69. Langan NP, & Pelissier, B. M. M. Gender differences among prisoners in drug treatment. *Journal of Substance Abuse.* 2001;13(3):291-301.

70. Pelissier B. Gender differences in substance use treatment entry and retention among prisoners with substance use histories. *American Journal of Public Health.* 2004;94(8):1418-1424.
71. Harlow C. *Education and Correctional Populations.* Bureau of Justice Statistics;2003.
72. Mann JJ, Apter A, Bertolote J, et al. Suicide prevention strategies: a systematic review. *JAMA : the journal of the American Medical Association.* 2005;294(16):2064-2074.
73. Kapur N, Cooper J, Bennewith O, Gunnell D, Hawton K. Postcards, green cards and telephone calls: therapeutic contact with individuals following self-harm. *The British journal of psychiatry : the journal of mental science.* 2010;197(1):5-7.
74. Vaiva G, Ducrocq F, Meyer P, et al. Effect of telephone contact on further suicide attempts in patients discharged from an emergency department: randomised controlled study. *BMJ.* 2006;332(7552):1241-1245.
75. Fleischmann A, Bertolote JM, Wasserman D, et al. Effectiveness of brief intervention and contact for suicide attempters: a randomized controlled trial in five countries. *Bulletin of the World Health Organization.* 2008;86(9):703-709.
76. Motto JA, Bostrom AG. A randomized controlled trial of postcrisis suicide prevention. *Psychiatr Serv.* 2001;52(6):828-833.
77. Carter GL, Clover K, Whyte IM, Dawson AH, D'Este C. Postcards from the EDge: 5-year outcomes of a randomised controlled trial for hospital-treated self-poisoning. *The British journal of psychiatry : the journal of mental science.* 2013;202(5):372-380.
78. Carter GL, Clover K, Whyte IM, Dawson AH, D'Este C. Postcards from the EDge: 24-month outcomes of a randomised controlled trial for hospital-treated self-poisoning. *The British journal of psychiatry : the journal of mental science.* 2007;191:548-553.
79. Carter GL, Clover K, Whyte IM, Dawson AH, D'Este C. Postcards from the EDge project: randomised controlled trial of an intervention using postcards to reduce repetition of hospital treated deliberate self poisoning. *BMJ.* 2005;331(7520):805.
80. Jobes DA. The Collaborative Assessment and Management of Suicidality (CAMS): an evolving evidence-based clinical approach to suicidal risk. *Suicide & life-threatening behavior.* 2012;42(6):640-653.
81. Ramsay R. New developments in suicide intervention training. *Suicidologi.* 2004;9(3):10-12.
82. Linehan MM, Comtois KA, Murray AM, et al. Two-year randomized controlled trial and follow-up of dialectical behavior therapy vs therapy by experts for suicidal behaviors and borderline personality disorder. *Archives of general psychiatry.* 2006;63(7):757-766.
83. Johnson JE, Schonbrun, Y. C., Peabody, M. E., Shefner, R. T., Fernandes, K. M., Rosen, R. K., & Zlotnick, C. . Provider experiences with prison care and aftercare for women with co-occurring mental health and substance use disorders: Treatment, resource and systems integration challenges. under review.
84. Wolff N. Community reintegration of prisoners with mental illness: A social investment perspective. *International Journal of Law and Psychiatry.* 2005;28:43-58.
85. Travis J, Petersilia, J. . Re-entry reconsidered: A new look at an old question. *Crime & Delinquency.* 2001;47:291-313.
86. Peters R, LeVasseur, ME, Chandler, RK. Correctional treatment for co-occurring disorders: Results of a national survey. *behavioral Sciences and the Law.* 2004;22:563-584.

87. Human_Rights_Watch. *Ill-equipped: U.S. prisons and offenders with mental illness.* Washington, DC: Human Rights Watch;2003.
88. VanderWaal CJ, Taxman, F.S., Gurka-Ndanyi, M.A. Reforming drug treatment services to offenders: Cross-system collaboration, integrated policies, and a seamless continuum of care model. *Journal of Social Work Practice in the Addictions.* 2008;8(1):127-153.
89. Daniel A. Care of the Mentally Ill in Prisons: Challenges and Solutions. *J Am Acad Psychiatry Law.* 2007;35:406-410.
90. Taxman FS, & Belenko, S. *Implementing Evidence-Based Practices in Community Corrections and Addiction Treatment.* New York: Springer; 2011.
91. Perdoni M, Taxman, F. S., & Fletcher, B. W. Treating offenders in the community: An overlooked population and a lost public health and public safety opportunity. *Perspectives.* 2008;32(2):46-53.
92. Stanley B, Brown GK. Safety Planning Intervention: A Brief Intervention to Mitigate Suicide Risk. *Cogn Behav Pract.* 2012;19(2):256-264.
93. Knox KL, Stanley B, Currier GW, Brenner L, Ghahramanlou-Holloway M, Brown G. An emergency department-based brief intervention for veterans at risk for suicide (SAFE VET). *American journal of public health.* 2012;102 Suppl 1:S33-37.
94. Stanley B, Brown GK. *The Safety Plan Treatment Manual to Reduce Suicide Risk: Veteran Version.* Washington, DC: United States Department of Veterans Affairs; 2008.
95. Ghahramanlou-Holloway M, Cox DW, Fritz EC, George BJ. An Evidence-Informed Guide for Working With Military Women and Veterans. *Prof Psychol-Res Pr.* 2011;42(1):1-7.
96. Ghahramanlou-Holloway M, Bhar SS, Brown GK, Olsen C, Beck AT. Changes in problem-solving appraisal after cognitive therapy for the prevention of suicide. *Psychological medicine.* 2012;42(6):1185-1193.
97. Salkovskis PM, Atha C, Storer D. Cognitive-behavioural problem solving in the treatment of patients who repeatedly attempt suicide. A controlled trial. *The British journal of psychiatry : the journal of mental science.* 1990;157:871-876.
98. Bryan CJ, Hernandez AM. The functions of social support as protective factors for suicidal ideation in a sample of air force personnel. *Suicide & life-threatening behavior.* 2013;43(5):562-573.
99. Pietrzak RH, Goldstein MB, Malley JC, Rivers AJ, Johnson DC, Southwick SM. Risk and protective factors associated with suicidal ideation in veterans of Operations Enduring Freedom and Iraqi Freedom. *Journal of affective disorders.* 2010;123(1-3):102-107.
100. Poudel-Tandukar K, Nanri A, Mizoue T, et al. Social support and suicide in Japanese men and women - the Japan Public Health Center (JPHC)-based prospective study. *Journal of psychiatric research.* 2011;45(12):1545-1550.
101. Rowe JL, Conwell Y, Schulberg HC, Bruce ML. Social support and suicidal ideation in older adults using home healthcare services. *The American journal of geriatric psychiatry : official journal of the American Association for Geriatric Psychiatry.* 2006;14(9):758-766.
102. Beautrais AL. Effectiveness of barriers at suicide jumping sites: a case study. *Aust Nz J Psychiat.* 2001;35(5):557-562.
103. Beautrais AL, Fergusson DM, Horwood LJ. Firearms legislation and reductions in firearm-related suicide deaths in New Zealand. *Aust Nz J Psychiat.* 2006;40(3):253-259.

104. Daigle MS. Suicide prevention through means restriction: Assessing the risk of substitution - A critical review and synthesis. *Accident Anal Prev.* 2005;37(4):625-632.
105. Hawton K. United Kingdom legislation on pack sizes of analgesics: Background, rationale, and effects on suicide and deliberate self-harm. *Suicide Life-Threat.* 2002;32(3):223-229.
106. Hawton K. UK legislation on analgesic packs: before and after study of long term effect on poisonings (vol 329, pg 1076, 2004). *Brit Med J.* 2004;329(7475):1159-1159.
107. Stanley B, Brown, G. Safety Planning Intervention: A brief intervention to mitigate suicide risk. *Cognitive and Behavioral Practice.* in press.
108. Jakupcak M, Varra EM. Treating Iraq and Afghanistan War Veterans With PTSD Who Are at High Risk for Suicide. *Cogn Behav Pract.* 2011;18(1):85-97.
109. Kress VE, Hoffman RM. Non-suicidal self-injury and motivational interviewing: Enhancing readiness for change. *Journal of Mental Health Counseling.* 2008;30:311-329.
110. McMurran M. Motivational interviewing with offenders: A systematic review. *Legal Criminol Psych.* 2009;14(1):83-100.
111. Stanley B. The Safety Planning Intervention and other brief interventions to mitigate risk with suicidal individuals. Texas Suicide Prevention Symposium; 2009; Austin, TX.
112. Battaglia L, Coleman MJ, Puorto Conte M, et al. Getting to the Goal: Suicide as a Never Event in New York State. In: Management DoQ, ed. Albany, NY: NY State Office of Mental Health; 2013.
113. Knox K, Brown G, Brenner L, Currier G, Stanley B. SAFE VET: A brief intervention to mitigate suicide risk in acute settings: Initial Findings. Paper presented at: VHA Mental Health Conference2011; Baltimore, MD.
114. Knox K, Brown G, Currier G, Stanley B. Brief interventions to mitigate suicide risk in acute settings: Initial findings and implications. Paper presented at: DoD/VA Annual Suicide Prevention Conference2011; Boston, MA.
115. Knox K, Stanley, B, Currier, G, Brenner, L, Ghahramanlou-Holloway, M, Brown, G. An emergency-department based brief intervention for veterans at risk for suicide (SAFE VET). *American Journal of Public Health.* 2012;102:s33-s37.
116. Alonzo DM, Harkavy-Friedman, J.M., Stanley, B., Burke, A., Mann, J. J., Oquendo, M.A. Predictors of treatment utilization in major depression. . *Archives of Suicide Research.* 2011;15:160-171.
117. Litman RE. Suicidology: A look backward and ahead. . *Suicide and Life-Threatening Behavior.* 1996;26:1-7.
118. Rihmer Z. Suicide risk in mood disorders. *Current Opinion in Psychiatry.* 2007;20:17-22.
119. Morgan R, Rozycki, AT, Wilson, S. Inmate perceptions of mental health services. *Professional Psychology: Research and Practice.* 2004;35(4):389-396.
120. Wormith JS, Althouse, R., Simpson, M., Reitzel, L.R., Fagan, T.J., Morgan, R.D. The rehabilitation and reintegration of offenders: The current landscape and future directions for correctional psychology. *Criminal Justice and Behavior.* 2007;34(7):879-892.
121. Howerton A, Byng, R., Campbell, J.. Hess, D., Owens, C., Aitken, P. . Understanding help seeking behaviour among male offenders: qualitative interview study. *BMJ: British Medical Journal.* 2007;344(7588):303.
122. Baillargeon J, Hoge, S.K., Penn, J.V. Addressing the challenge of community reentry among released inmates with serious mental illness. . *American Journal of Community Psychology.* 2010;46(3-4):361-375.

123. Haimowitz S, Appelbaum, PS. Slowing the revolving door: Community reentry of offenders with mental illness. *Psychiatric SErvices.* 2004;55(4):373-375.

124. Mallik-Kane K, Visher, C.A. *Health and Prisoner Reentry: How Physical, Mental, and Substance Abuse Conditions Shape the Process of Reintegration.* Urban Institute: Justice Policy Center;2008.

125. US Department of Health and Human Services Special Projects of National Significance. Enhancing linkages and access to care in jails. *What's going on at Special Projects of National Significance* July 2012; http://hab.hrsa.gov/abouthab/files/cyberspnsjuly12.pdf.

126. Veysey B, Steadman, HJ, Morrissey, JP, Johnsen, M. In search of the missing linkages: Continuity of care in U.S. jails. *Behav. Sci. Law.* 1997;15(4):383-397.

127. Young DW, Farrell, J. L., Henderson, C. E., & Taxman, F. S. Filling service gaps: Providing intensive treatment services for offenders. *Drug and Alcohol Dependence.* 2009;103(Suppl. 1):S33-S42.

128. Young D. Co-occurring disorders among jail inmates: Bridging the treatment gap. *Journal of Social Work Practice in the Addictions.* 2003;3(3):63-85.

129. Grella CE, Rodriguez, L. . Motivation for treatment among women offenders in prison-based treatment and longitudinal outcomes among those who participate in community aftercare. *Journal of Psychoactive Drugs.* 2011;Sarc Suppl 7:58-67.

130. McMurran M. Notivational interviewing with offenders: A systematic review. *Legal and Criminological Psychology.* 2009;14:83-100.

131. SAMHSA. *Substance Abuse Treatment for Adults in the Criminal Justice System: A Treatment Improvement Protocol (TIP 44).* Vol DHHS Publication No. (SMA) 05-4056. Rockville, MD: SAMHSA; 2005.

132. Johnson J. Integrating psychotherapy research with public health and public policy goals for incarcerated women and other vulnerable populations. *Psychotherapy Research.* in press.

133. Johnson JE, Schonbrun, Y. C., & Stein, M. D. Pilot test of twelve-step linkage for alcohol abusing women leaving jail. . *Substance Abuse.* 2013;DOI: 10.1080/08897077.2013.794760.

134. Biggam F, Power, KG. A comparison of the problem-solving abilities and psychological distress of suicidal, bullied, and protected prisoners. *Criminal Justice and Behavior.* 1999;1999(2):196-216.

135. Hayward JM, M., Sellen, J. Social problem solving in vulnerable adult prisoners: Profile and intervention. *Forensic Psychiatry & Psychology.* 2008;19(2):243-248.

136. Ivanoff A, Smyth, NJ, Grochowski, S, Jang, SJ, Klein, KE. Problem solving and suicidality among prison inmates: Another look at state vs. trait. *Journal of Consulting and Clinical Psychology.* 1992;60(6):970-973.

137. Bonner RL, Rich A. Negative Life Stress, Social Problem-Solving Self-Appraisal, and Hopelessness - Implications for Suicide Research. *Cognitive Ther Res.* 1988;12(6):549-556.

138. Pollock LR, Williams JM. Problem-solving in suicide attempters. *Psychological medicine.* 2004;34(1):163-167.

139. Rudd MD, Rajab MH, Dahm PF. Problem-solving appraisal in suicide ideators and attempters. *The American journal of orthopsychiatry.* 1994;64(1):136-149.

140. Eidhim M, Sheehy, N, O'Sullivan, M, McLeavey, B. Perceptions of the environment, suicidal ideation and problem-solving deficits in an offender population. *Legal and Criminological Psychology.* 2002;7:187-201.
141. Shanteau J, Dino, GA. Environmental stressor effects on creativity and decision making. In: Svenson A, Maul, AJ, ed. *Time Pressure and Stress in Human Judgment and Decision Making*. New York: Plenum Press; 1993:293-309.
142. Byron K, Khanzanchi, S., Nazarian, D. The relationship between stressors and creativity: A meta-analysis examining competing theoretical models. *Journal of Applied Psychology*. 2010;95(1):201-212.
143. Porcelli A, Delgado, MR. Acute stress modulates risk taking in financial decision making. *Psychological Science.* 2009;20(3):278-283.
144. Van Orden KA, Witte TK, Cukrowicz KC, Braithwaite SR. The interpersonal theory of suicide. *Psychological Review.* 2010;117:575-600.
145. Van Orden K, Witte, TK, Gordon, KH, Bender, TW, Joiner, TE Jr. Suicidal desire and the capability for suicide: Tests of the interpersonal-psychological theory of suicidal behavior among adults. *Journal of Consulting and Clinical Psychology.* 2008;76:72-83.
146. Dexter P, Towl, G. An investigation into suicidal behaviours in prison. *Issues in Criminological & Legal Psychology.* 1995;22:45-53.
147. Way B, Miraglia, R, Sawyer, DA, Beer, R, Eddy, J. Factors related to suicide in New York state prisons. *International Journal of Law and Psychiatry.* 2005;28:207-221.
148. Bonner R, Rich, AR. Psychosocial vulnerability, life stress, and suicide ideation in a jail population: A cross-validation study. *Suicide and Life-Threatening Behavior.* 1990;20(3):213-224.
149. Brown S, Day, A. The role of loneliness in prison suicide prevention and management. *Journal of Offender Rehabilitation.* 2008;47(4):433-449.
150. Johnson J. W, C., & Zlotnick C. Development and pilot testing of a cellphone-based transitional intervention for women prisoners with comorbid substance use and depression. . *The Prison Journal.* in press.
151. Johnson J. Integrating psychotherapy research with public health and public policy goals for incarcerated women and other vulnerable populations. *Psychotherapy Research.* 2014;24(2):229-239.
152. Johnson J, Schonbrun, YC, Peabody, ME, Shefner, RT, Fernandes, KM, Rosen, RK, Zlotnick, C. Provider experiences with prison care and aftercare for women with co-occurring mental health and substance use disorder: Treatment, resource, and systems integration challenges. *Journal of Behavioral Health Services & Research.* in press.
153. National Action Alliance for Suicide Prevention: Research Prioritization Task Force. A prioritized research agenda for suicide prevention: An action plan to save lives. 2014; www.suicide-research-agenda.org.
154. Richardson J, Mark, TL, McKeon. The return on investment of postdischarge follow-up calls for suicidal ideation or deliberate self-harm. *Psychiatric Services.* 2014;65(8):1012-1019.
155. Asgard U, Carlsson-Berjstrom M. Interviews with survivors of suicides: procedures and follow-up of interview subjects. *Crisis.* 1991;12(1):21-33.
156. Catanzaro R, Green W. WATS telephone therapy: new follow-up technique for alcoholics. *American J. of Psychiatry.* 1970;126(7):148-151.

157. Cuijpers P. Bibliotherapy in unipolar depression: a meta analysis. *Journal of Behavior Therapy and Experiemental Psychiatry.* 1997;28:139-147.
158. Grumet G. Telephone therapy: a review and case report. *Amer. J. of Orthopsychiatry.* 1979;49(4):574-584.
159. Horng FF, Chueh KH. Effectiveness of telephone follow-up and counseling in aftercare for alcoholism. *J Nurs Res.* 2004;12(1):11-20.
160. Intagliata J. A telephone follow-up procedure for increasing the effectiveness of a treatment program for alcoholics. *J. of Studies on Alcohol.* 1976;37(9):1330-1335.
161. Johnson MJ, Frank DI. Effectiveness of a telephone intervention in reducing anxiety of families of patients in an intensive care unit. *Appl Nurs Res.* 1995;8(1):42-43.
162. Lane AB. Combining telephone peer counseling and professional services for clients in intensive psychiatric rehabilitation. *Psychiatr Serv.* 1998;49(3):312-314.
163. Mahoney DF, Tarlow BJ, Jones RN. Effects of an automated telephone support system on caregiver burden and anxiety: findings from the REACH for TLC intervention study. *Gerontologist.* 2003;43(4):556-567.
164. Meyersburg G. The use of the telephone in psychiatric rehabilitation. *Nordisk Psykiatrisk Tidsskrift.* 1985;39(3):185-188.
165. Moreland H, Grier M. Telephone consultation in the care of older adults. *Geriatric Nursing.* 1986:28-30.
166. Nelson E, Van Cleeve S, Swartz M, Kessen W, McCarthy P. Improving the use of early follow-up care after emergency department visits. *American J. of Diseases of Children.* 1991;145:440-444.
167. Orleans C, Shoenback V, Wagner E, et al. Self-help quit smoking interventions: effects of self-help materials, social support instructions, and telephone counseling. *J. of Consulting & Clinical Psychology.* 1991;59(3):439-448.
168. Osgood-Hynes DJ, Greist JH, Marks IM, et al. Self-administered psychotherapy for depression using a telephone-accessed computer system plus booklets: an open U.S.-U.K. study. *J Clin Psychiatry.* 1998;59(7):358-365.
169. Parker JD, Turk CL, Busby LD. A brief telephone intervention targeting treatment engagement from a substance abuse program wait list. *J Behav Health Serv Res.* 2002;29(3):288-303.
170. Skipwith D. Telephone counseling interventions with caregivers of elders. *Journal of Psychosocial Nursing.* 1994;32:7-12.
171. Tolchin J. Telephone psychotherapy with adolescents. *Adolscent Psychiatry.* 1987;14:332-341.
172. Tolchin J. Telephone psychotherapy with adolescents. 1987.
173. Bishop D, Miller I. Development of a Family Telephone Intervention for Stroke Patients. NIMH Grant; 1995-8.
174. Bishop D, Miller I, Weiner D, Albro J. Telephone administered family intervention following stroke. submitted for publication.
175. Miller I. Efficacy of a Family Telephone Intervention for Stroke. *NINDS Grant NS37840.* 1998.
176. Stein M. Adapting to HIV Disease - A Family Intervention. *NIMH Grant MH63051.* 2000.
177. Tremont G. A Telephone Intervention for Dementia Caregivers. *NIMH Grant MH62561.* 2001.

178. Kubiak SP, Zeoli, A.M., Essenmacher, L., Hanna, J. Transitions between jail and community-based treatment for individuals with co-occurring disorders. *Psychiatr Serv.* 2011;62(2):679-681.
179. McLearan A, Ryba, NL. Identifying severely mentally ill inmates: Can small jails comply with detection standards? *Journal of Offender Rehabilitation.* 2003;37(1):25-40.
180. Szykula SA, Jackson, Dawn F. Managed Mental Health Care in Large Jails: Empirical Outcomes on Cost and Quality. *Journal of Correctional Health Care.* 2005;11(3):223-240.
181. Posner K, Brown GK, Stanley B, et al. The Columbia-Suicide Severity Rating Scale: initial validity and internal consistency findings from three multisite studies with adolescents and adults. *The American journal of psychiatry.* 2011;168(12):1266-1277.
182. Keller M, Lavori P, Friedman B, et al. The Longitudinal Interval Follow-up Evaluation. 1987;44:540-548.
183. Burdine JN, Felix MRJ, Abel AL, Wiltraut CJ, Musselman YJ. The SF-12 as a population health measure: An exploratory examination of potential for application. *Health Serv Res.* 2000;35(4):885-904.
184. Linehan MM, Heard HL. Treatment history interview (THI). Seattle, WA: University of Washington; 1987.
185. Freedenthal S, Lamis DA, Osman A, Kahlo D, Gutierrez PM. Evaluation of the psychometric properties of the Interpersonal Needs Questionnaire-12 in samples of men and women. *Journal of clinical psychology.* 2011;67(6):609-623.
186. Van Orden KA, Cukrowicz KC, Witte TK, Joiner TE. Thwarted Belongingness and Perceived Burdensomeness: Construct Validity and Psychometric Properties of the Interpersonal Needs Questionnaire. *Psychol Assessment.* 2012;24(1):197-215.
187. Sheehan D, Lecrubier Y, Sheehan K, et al. The Mini-International Neuropsychiatric Interview (M.I.N.I): the development and validation of a structured diagnostic psychiatric interview for DSM-IV and ICD-10. *J. Clin. Psychiatry.* 1998;59(suppl 20):22-33.
188. Posner K, Oquendo MA, Gould M, Stanley B, Davies M. Columbia Classification Algorithm of Suicide Assessment (C-CASA): classification of suicidal events in the FDA's pediatric suicidal risk analysis of antidepressants. *The American journal of psychiatry.* 2007;164(7):1035-1043.
189. Linehan M, Heard H. *Treatment History Interview-4 (THI-4).* Seattle,WA: University of Washington; 1996.
190. Keller MB, Lavori PW, Friedman B, et al. The Longitudinal Interval Follow-up Evaluation. A comprehensive method for assessing outcome in prospective longitudinal studies. *Archives of general psychiatry.* 1987;44(6):540-548.
191. Sibrava N, Beard, C, Bjornsson, AS, Moitra, E, Weisberg, RB, Keller, MB Two-year course of generalized anxiety disorder, social anxiety disorder, and panic disorder in a longitudinal sample of African American adults. *Journal of Consulting and Clinical Psychology.* 2013;81(6):1052-1062.
192. Bjornsson A, Sibrava, NJ, Beard, C, Moitra, E, Weisberg, RB, Perez Benitez, CI, Keller, MB Two-year course of generalized anxiety disorder, social anxiety disorder, and panic disorder with agoraphobia in a sample of Latino adults. *Journal of Consulting and Clinical Psychology.* 2014;Online First Publication, April 14, 2014. http://dx.doi.org/10.1037/a0036565.

193. Van Orden KA, Cukrowicz KC, Witte TK, Joiner TE. Thwarted belongingness and perceived burdensomeness: construct validity and psychometric properties of the Interpersonal Needs Questionnaire. *Psychol Assess.* 2012;24(1):197-215.

194. Boudreaux E, Miller, I, Goldstein, AB, Sullivan, AF, Allen, MH, Manton, AP, Arias, SA, Camargo, CA. The Emergency Department Safety Assessment and Follow-up Evaluation (ED-SAFE): Methods and design considerations. *Contemporary Clinical Trials.* 2013;36(1):14-24.

195. Arias S, Zhang, Z, Hillerns, C, Sullivan, AF, Boudreaux, ED, Miller, IW, Camargo, CA. Using structured telephone follow-up assessments to improve suicide-related adverse event detection. . *Suicide and Life-Threatening Behavior.* 2014;44:537-547. PMCID in progress.

196. CDC_National_Center_For_Health_Statistics. *National Death Index user's guide.* Hyattsville, MD2013.

197. Miller TR, Taylor, D.M. Adolescent suicidality: Who will ideate, who will act? *Suicide and Life-Threatening Behavior.* 2005;35(4):425-435.

198. Sengupta N, Nichol, M.B., Wu, J., Globe, D. . Mapping the SF-12 to the HUI3 and VAS in a managed care population. *Med Care.* 2004;42(9):927-937.

199. Gold M, Siegel, JE, Russell, LB, Weinstein, MC, ed *Cost-Effectiveness in Health and Medicine.* New York: Oxford University Press; 1996.

200. Kraemer H, Morgan, GA, Leech, NL, Gliner, JA, Vaske, JJ, Harmon, RJ. Measures of Clinical Significance. *Journal of the American Academy of Child & Adolescent Psychiatry.* 2003;42(12):1524-1529.

201. CONSORT. CONSORT: Transparent reporting of trials. 2010; http://www.consort-statement.org/.

202. Little R, Rubin D. *Statistical Analysis with Missing Data.* New York: John Wiley and Sons; 1987.

203. Allison PD. *Missing Data.* Thousand Oaks, CA: Sage Publications; 2002.

204. Simons J, Neal, DJ, Gaher, RM. Risk for marijuana-related problems among college students: An application of zero-inflated negative binomial regression. *The American Journal of Drug and Alcohol Abuse.* 2006;32:41-53.

205. Walters G. Using Poisson class regression to analyze count data in correctional and forensic psychology: A relatively old solution to a relatively new problem. *Criminal Justice and Behavior.* 2007;34(12):1659-1674.

206. MacKinnon DP, Lockwood CM, Hoffman JM, West SG, Sheets V. A comparison of methods to test mediation and other intervening variable effects. *Psychol Methods.* 2002;7(1):83-104.

207. Neumann P. *Using cost-effectiveness analysis to improve health care.* New York: Oxford University Press; 2005.

208. Meckley LM, Greenberg, D., Cohen, J.T., Neumann, P.J. The Adoption of Cost-Effectiveness Acceptability Curves in Cost-Utility Analyses. *Med Decis Making.* 2010;30(3):314-319.

209. Guthrie E, Kapur, N, Mackway-Jones, K, Chew-Graham, C, Moorey J, Mendel, E, Marino-Francis, F, Sanderson, S, Turpin, C, Boddy, G, Tomenson, B. Randomised controlled trial of brief psychological intervention after deliberate self poisoning. *BMJ.* 2001;323(7305):135.

210. Kraemer HC, Morgan GA, Leech NL, Gliner JA, Vaske JJ, Harmon RJ. Measures of clinical significance. *J. Am. Acad. Child Adolesc. Psychiatry*. 2003;42(12):1524.

211. Vittinghoff E, Sen Ś, McCulloch C. Sample size calculations for evaluating mediation. *Stat. Med.* 2009;28(4):541-557.

21. Attachments/Appendices

(1) Informed consent document with locator form and release of information for medical records review (local hospital and jail records)

(2) Safety Planning Intervention manual and fidelity rating scale

(3) Overview of participant flow through study (Figure)

186 **DSMB protocol chang es:**

Protocol Version#	Description of amendment and pages amended
2	P.6,19,24,35 The study has obtained a national Institute of Justice (NIJ) Privacy Certificate instead of a Certificate of Confidentiality.<br>P.6,29-30 Edits to the assessment protocol (1) removed MINI PTSD module and replaced with Life Events Checklist/PTSD Checklist (PCL); (2) removed DAST-10 and replaced with Drug Use Identification Test (DUDIT); (3) removed the SF-12 for the VR-12; (4) add the KG screening instrument for severe psychopathology.<br>P.7,15-16,19-22,24,26-27,29-30,32-33 Study timeline for follow-up assessments will be specified in weeks (vs. months). As such, research assessment time points were re-specified to occur at 4 weeks, 16 weeks, 34 weeks, and 52 weeks post-release from jail (vs. 1 month, 4 months, 8 months, and 12 months.<br>P.18 Updated anticipated enrollment table.<br>P.18-19,23 There will not be separate consents for the screening phase of the study and the main study. All who meet basic screening requirements will be consented. After consenting, the Research Assistant will continue with the assessments to determine whether the detainee meets full criteria for randomization and full participation in the study.<br>P.23 Add the option for jail or research staff to introduce the study to inmates.<br>P.36 Add Co-Investigator to the study to assist with AE/SAE reporting and preparation of DSMB reports.<br>P.44 Modify the AE/SAE reporting protocol to specify that we will only report unexpected events to the- IRB. The AE/SAE reporting protocol for the NIMH DSMB has not been modified.
3	P.15,34 Expand inclusion criteria to specify threshold of suicide risk as response of 4+ on C-SSRS screener items OR a suicide attempt in the 30 days prior to baseline.
4	P.19 Optional intervention calls increase from 2 to 4 calls based on clinical need.<br>P.24 Reporting inappropriate behavior towards study staff to the jail staff.<br>P.28 Update Boys Town transfer guideline definitions for emergent and non-emergent suicidal ideation or behavior.<br>P.30 Add brief questionnaire for feedback about the study.<br>P.44 Revised wording to specify that only unanticipated and unexpected SAEs (including deaths will be rep orted to IRB p er their , procedures. ---
5	P.12,25,47 Replace regular recorders with encrypted recorders per- IRB request.<br>PP.15-16 If a participant is sentenced to more than 30 days in jail or goes directly to jail, s/he is ineligible.<br>P.16,19-20,25,27,28,30 Language added to include option to have in-person or phone interviews if participant is in a residential treatment facility.<br>P.19 Add middle name and city/municipality of birth to consent locator forms to meet NIH data sharing guidelines.<br>P.49 Pay particip ants with a gift card if they do not have an ID to cash a money order.
6	P.43 Modify site monitoring plan from both sites every 6 months to alternating sites every 6 months, in addition to periodic site visits from IRB --
7	P.50 Language was added to clarify that if the participant is incarcerated in the [redacted], s/he is not eligible for compensation per [redacted] reg ulations.
8	P.44 Revised definitions of "expected" and "unexpected" adverse events in response to DSMB req uest.
9	Added information from the administrative supplement received in Aug ust 2018:

	P.17 Language included to indicate that recruitment was extended through November 2018.<br>P.29-30 Measures added to outcome section for overdose and Safety plan use in the context of substance use.<br>P. 32 Data analysis section updated to include additional analyses from supplement.
10	P.16 Table revised to clarify when PIs are blinded to outcome data.<br>P.32 Timeline added to include information from the original grant text. Timeline clarifies when we will close the assessment windows and begin data analysis for each wave of data.<br>P.34 Text added to clarify what will happen to participants who are not released from jail by assessment window closing date.
11	P.32 Updated timeline that we will close the assessment window for the 52-week assessment.

187
