# Supplementary material for: Safety Planning vs Standard Care for Suicide Prevention After Pretrial Jail Detention: A Randomized Clinical Trial
Source: JAMA Netw Open. 2025 Nov 10;8(11):e2543156. doi: 10.1001/jamanetworkopen.2025.43156 (PMC12603856; doi:10.1001/jamanetworkopen.2025.43156)
Supplement: Supplement 2. — eTable 1. Distribution of Hypothesized Mediators and Differences by Treatment Group eTable 2. Treatment Effect Mediation eTable 3. Sensitivity Analysis: Winsorized Cumulative Suicide Events and Cumulative Suicide Attempts Observed Over up to 12 Months After Jail Release, and Differences by Treatment Group, Adjusted eFigure. Distribution of Hypothesized Mediators Observed Over Follow-Up According to Treatment Group [file jamanetwopen-e2543156-s002.pdf]

Supplemental Online Content

Weinstock LM, Jones RN, Miller TR, et al. Safety planning vs standard care for suicide prevention after pretrial jail detention: a randomized clinical trial. *JAMA Netw Open*. 2025;8(1):e2543156. doi:10.1001/jamanetworkopen.2025.43156

eTable 1. Distribution of Hypothesized Mediators and Differences by Treatment Group
eTable 2. Treatment Effect Mediation
eTable 3. Sensitivity Analysis: Winsorized Cumulative Suicide Events and Cumulative Suicide Attempts Observed Over up to 12 Months After Jail Release, and Differences by Treatment Group, Adjusted
eFigure. Distribution of Hypothesized Mediators Observed Over Follow-Up According to Treatment Group

This supplemental material has been provided by the authors to give readers additional information about their work.

© 2025 Weinstock LM et al. *JAMA Network Open*.

eTable 1. Distribution of Hypothesized Mediators and Differences by Treatment Group

Outcome	ESC (N=313)		SPI (N=342)		Difference		
	Mean	(SE)	Mean	(SE)	Est	(SE)	P
Treatment utilization (number of outpatient mental health and substance use visits)	8.41	(0.86)	9.02	(0.88)	0.61	(1.23)	.62
Suicide-related problem solving	65.39	(0.69)	65.82	(0.66)	0.43	(0.96)	.65
Belongingness	20.08	(0.81)	19.90	(0.77)	-0.19	(1.12)	.87

Note: ESC; Enhanced Standard Care; SPI, Safety Planning Intervention with telephone follow-up; Mean, marginal mean; SE, standard error; P, p-value. Marginal means, standard errors, and differences according to treatment group from negative binomial (Treatment utilization) or linear regression models (Suicide-related problem solving, belongingness) controlling for baseline value of the mediator and sex. Treatment utilization was measured using the Treatment History Interview combined with medical records review. Suicide-related problem solving was measured using the Suicide Related Coping Scale. Belongingness was measured using the corresponding subscale of the Interpersonal Needs Questionnaire.

© 2025 Weinstock LM et al. *JAMA Network Open*.

eTable 2. Treatment Effect Mediation

Table entries present the percent of treatment effect explained by target mediator(s).

Mediator	Pct of Treatment Effect Explained, *Composite Suicide Behaviors and Attempts*	Pct of Treatment Effect Explained, *Cumulative Suicide Attempts*
Treatment utilization	-1.9	1.8
Suicide-related problem solving	9.2	4.2
Belongingness	5.3	0.5
All 3 mediators together	9.7	9.8

Note: Table entries represent the percent change (pct) in the Safety Planning Intervention with telephone follow-up (SPI) treatment effect after adjusting for each candidate mediator. Values are calculated as: $100\times(1-b')/b$ where b is the treatment effect from the base model and b' is the effect after adjusting for the mediator. A positive value indicates that the mediator explains part of the treatment effect (i.e., consistent with mediation). A negative value indicates that the treatment effect increases when the mediator is included—suggesting suppression. We interpret reductions of ≥10% as meaningful, following the change-in-estimate criterion proposed by Maldonado and Greenland (1993). Treatment utilization was measured using the Treatment History Interview combined with medical records review. Suicide-related problem solving was measured using the Suicide Related Coping Scale. Belongingness was measured using the corresponding subscale of the Interpersonal Needs Questionnaire.

See text for additional details.

© 2025 Weinstock LM et al. *JAMA Network Open*.

eTable 3. Sensitivity Analysis: Winsorized Cumulative Suicide Events and Cumulative Suicide Attempts Observed Over up to 12 Months After Jail Release, and Differences by Treatment Group, Adjusted

**Outcome**	**ESC (N=313)**		**SPI (N=342)**		**Difference**		
	**Mean**	**(SE)**	**Mean**	**(SE)**	**Est**	**(SE)**	**P**
Cumulative suicide events, Winsorized	2.12	(0.19)	1.67	(0.14)	-0.45	(0.23)	.060
Cumulative suicide attempts, Winsorized	1.21	(0.14)	0.88	(0.10)	-0.33	(0.17)	.046

Notes: ESC, Enhanced Standard Care; SPI, Safety Planning Intervention with telephone follow-up; Mean, marginal mean; SE, standard error; P, p-value. Outcome and baseline predictor was Winsorized at the 95th percentile. Estimated means reflect adjustment for baseline value of the outcome variable (Table 2) or baseline composite suicide attempts, sex, and randomly assigned treatment group. Results derive from a negative binomial regression model.

© 2025 Weinstock LM et al. *JAMA Network Open*.

eFigure. Distribution of Hypothesized Mediators Observed Over Follow-Up According to Treatment Group

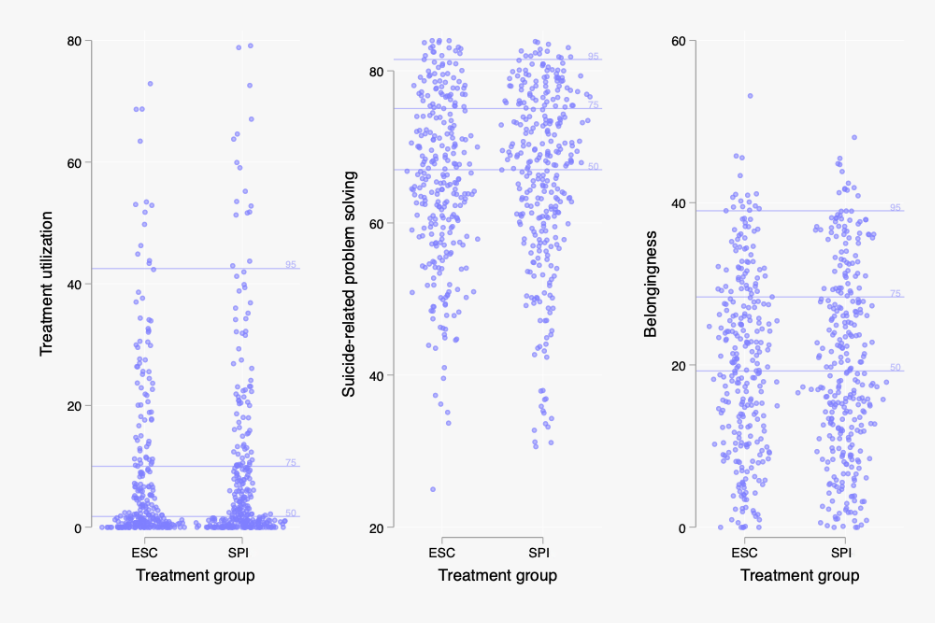

Note: ESC, Enhanced Standard Care; SPI, Safety Planning Intervention with telephone follow-up. Treatment utilization was measured using the Treatment History Interview combined with medical records review. Suicide-related problem solving was measured using the Suicide Related Coping Scale. Belongingness was measured using the corresponding subscale of the Interpersonal Needs Questionnaire. Distribution of mediators according to treatment group. Overall 50th, 75th, ad 95th percentiles are noted.

© 2025 Weinstock LM et al. *JAMA Network Open*.
