# Supplementary material for: Safety Planning vs Standard Care for Suicide Prevention After Pretrial Jail Detention: A Randomized Clinical Trial
Source: JAMA Netw Open. 2025 Nov 10;8(11):e2543156. doi: 10.1001/jamanetworkopen.2025.43156 (PMC12603856; doi:10.1001/jamanetworkopen.2025.43156)
Supplement: Supplement 3. — Data Sharing Statement [file jamanetwopen-e2543156-s003.pdf]

Data Sharing Statement

Weinstock. Safety Planning vs Standard Care for Suicide Prevention After Pretrial Jail Detention. *JAMA Netw Open*. Published November 13, 2025.
doi:10.1001/jamanetworkopen.2025.43156

Data

Additional Information: https://clinicaltrials.gov/study/NCT02759172
Data available: Yes
Data types: Deidentified participant data, Data dictionary
How to access data: NIMH National Data Archive: https://nda.nih.gov/
When available: beginning date: 07-01-2022

Supporting Documents

Document types: Other (please specify)
Additional Information: Available as supplements (submitted) with the manuscript.
How to access documents: None.
When available: With publication

Additional Information

Who can access the data: Anyone with access the NDA.
Types of analyses: For any purpose.
Mechanisms of data availability: Through the publicly available NDA.
